# An altered balance of integrated and segregated brain activity is a marker of cognitive deficits following sleep deprivation

**DOI:** 10.1371/journal.pbio.3001232

**Published:** 2021-11-04

**Authors:** Nathan E. Cross, Florence B. Pomares, Alex Nguyen, Aurore A. Perrault, Aude Jegou, Makoto Uji, Kangjoo Lee, Fatemeh Razavipour, Obaï Bin Ka’b Ali, Umit Aydin, Habib Benali, Christophe Grova, Thien Thanh Dang-Vu

**Affiliations:** 1 PERFORM Centre, Concordia University, Montreal, Canada; 2 Center for Studies in Behavioral Neurobiology, Department of Health, Kinesiology and Applied Physiology, Concordia University, Montreal, Canada; 3 Institut Universitaire de Gériatrie de Montréal and CRIUGM, CIUSSS du Centre-Sud-de-l’Île-de-Montréal, Montreal, Canada; 4 Multimodal Functional Imaging Lab, Department of Physics, Concordia University, Montreal, Canada; 5 Multimodal Functional Imaging Lab, Biomedical Engineering Department, Neurology and Neurosurgery Department, McGill University, Montreal, Quebec, Canada; 6 Department of Radiology and Biomedical Imaging, Yale University School of Medicine, New Haven, Connecticut, United States of America; 7 Social, Genetic, and Developmental Psychiatry Centre, Institute of Psychiatry, Psychology, and Neuroscience, King’s College London, London, United Kingdom; University of Glasgow, UNITED KINGDOM

## Abstract

Sleep deprivation (SD) leads to impairments in cognitive function. Here, we tested the hypothesis that cognitive changes in the sleep-deprived brain can be explained by information processing within and between large-scale cortical networks. We acquired functional magnetic resonance imaging (fMRI) scans of 20 healthy volunteers during attention and executive tasks following a regular night of sleep, a night of SD, and a recovery nap containing nonrapid eye movement (NREM) sleep. Overall, SD was associated with increased cortex-wide functional integration, driven by a rise of integration within cortical networks. The ratio of within versus between network integration in the cortex increased further in the recovery nap, suggesting that prolonged wakefulness drives the cortex towards a state resembling sleep. This balance of integration and segregation in the sleep-deprived state was tightly associated with deficits in cognitive performance. This was a distinct and better marker of cognitive impairment than conventional indicators of homeostatic sleep pressure, as well as the pronounced thalamocortical connectivity changes that occurs towards falling asleep. Importantly, restoration of the balance between segregation and integration of cortical activity was also related to performance recovery after the nap, demonstrating a bidirectional effect. These results demonstrate that intra- and interindividual differences in cortical network integration and segregation during task performance may play a critical role in vulnerability to cognitive impairment in the sleep-deprived state.

## Abstract 2

Can the cognitive changes that result from sleep deprivation be explained by information processing within and between large-scale networks in the brain? This study shows that the ratio of within- vs between-network integration is tightly associated with deficits in cognitive performance.

## Introduction

The cognitive consequences of acute total sleep deprivation (SD) are substantial, negatively affecting a wide range of processes including attention, vigilance, and working memory [1,2]. Yet, the measurable impact of SD on cognition varies across individuals in a trait-like manner [3,4]. Investigating intraindividual changes in brain activity patterns has significant potential in understanding the interindividual effects of prolonged wakefulness on cognitive functioning. Previous studies have demonstrated the effects of acute total SD on brain activity measured with functional magnetic resonance imaging (fMRI) during the resting state, such as disrupted connectivity within and between large-scale cortical networks [5] and a reduction in network modularity [6]. However, very few studies have investigated the changes in brain connectivity during the performance of cognitive tasks.

Both localised brain regions and cortical networks display characteristic and independent (segregated) activity patterns during the performance of tasks [7,8]. However, even simple behaviours and cognitive processes require the coordination (integration) of information flows across systems distributed in the brain [9]. It has been proposed that efficient cognitive functioning is reflected through a balance of segregation and integration of information across brain networks [10,11].

Endogenous activity fluctuations in the human brain share a robust association with ongoing cognitive and perceptual processes [12,13], as they may interfere with the capacity for information processing. Importantly, both local and global fMRI activity fluctuates at a significantly greater magnitude in states of reduced arousal and vigilance [14], as well as following SD [5,15,16]. Therefore, in states of reduced arousal, an elevation in endogenous fluctuations across the cortex may affect the ability to integrate information between and within cortical networks, corresponding to the cognitive deficits experienced during these states. These deficits may also be driven by certain changes in subcortical regions, such as the thalamus, which is involved in both maintaining cortical arousal [17] and constraining the engagement of distributed neural assemblies within the cerebral cortex [18]. However, this has not been explored following experimental manipulation of arousal states, such as SD. Furthermore, SD-induced cognitive impairment partially improves with subsequent sleep [19]. Yet, it remains unclear whether this behavioural recovery is associated with a functional “recovery” of cortical networks after sleep.

Here, we assessed the principles of functional brain activity across various arousal states using simultaneous electroencephalography (EEG)–fMRI recording and an SD protocol. Participants were scanned both at rest and while performing cognitive tasks probing attention, executive control, and working memory, after both a regular night of sleep and 24 hours of SD. Additionally, we measured EEG–fMRI activity during a recovery nap containing nonrapid eye movement (NREM) sleep following SD, as well as during a post recovery nap (PRN) session when the rest and task sequences were repeated. Firstly, to quantify the functional interactions within and between cortical networks across vigilance states, we computed a measure of integration of fMRI activity [20,21] within and between functional networks across different hierarchical levels of a representation of the cortex. We then compared the changes in functional integration during task performance to the magnitude of cognitive impairment following SD. In addition, we measured the association between recovery of both performance and the integration of cortical networks following the nap. Next, we assessed these changes in the context of changes to the amplitude of both local activity fluctuations and the global brain signal. Finally, we investigated the influence of thalamocortical activity on functional integration in the cortex across different arousal states.

## Results

**Fig 1:**
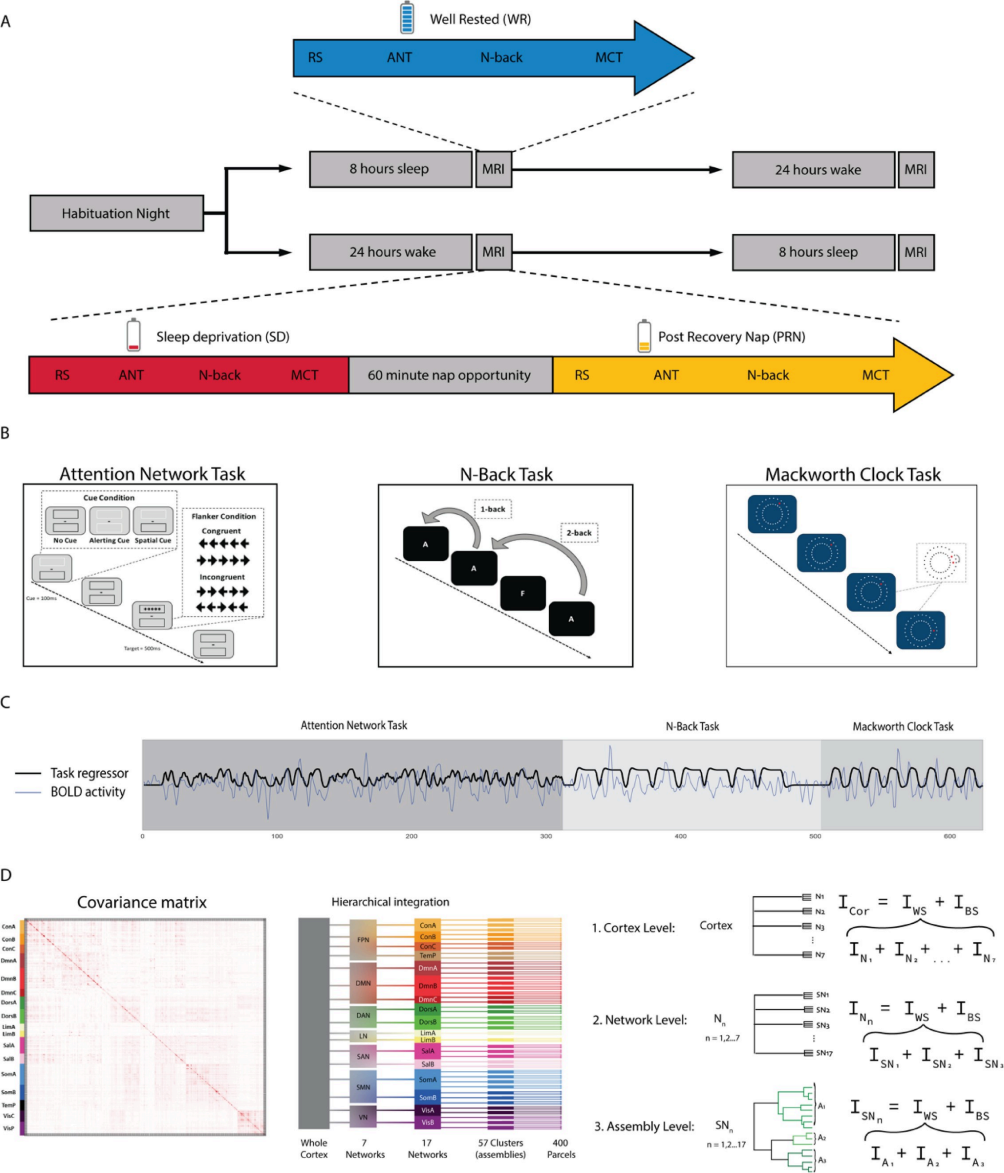
Study design and behavioural results. **(A)** Participants made 3 visits to the lab: a habituation night, followed by a counterbalanced design of either another full-night opportunity to sleep (blue) or a night of total SD. In the morning following each night, participants completed RS and cognitive tasks inside the MRI scanner. In the sleep-deprived state (red), participants also had a recovery nap opportunity (yellow) and then recompleted the tasks inside the MRI scanner. **(B)** Participants completed 3 cognitive tasks inside the scanner in each condition: the ANT, N-back task, and MCT. **(C)** The preprocessed BOLD time series was concatenated across the 3 tasks, providing one time series for each subject and condition. Task-specific activity (trials for the ANT, blocks for the N-back, and MCT) was modelled with a finite impulse response combined with a hemodynamic response function (black line) and regressed from the time series (blue line) for each parcel to reduce the influence of task-evoked coactivation in subsequent connectivity analyses. **(D)** Integration was calculated from the covariance matrix of cortical parcels, in a hierarchical manner: across the whole cortex, 7 networks, 17 networks, and 57 clusters. At the cortex level, total integration was calculated as the sum of integration within each of Yeo7 networks and a single measure of integration between networks (between systems). The total integration within each Yeo7 network was calculated as the sum of integration within each of Yeo17 subnetworks and the integration within each of the Yeo17 networks as the sum of integration within each cluster of parcels, as well as a single measure of integration between subnetworks or clusters. ANT, attention network task; BOLD, blood oxygen level dependent; MCT, Mackworth clock task; MRI, magnetic resonance imaging; RS, resting state; SD, sleep deprivation.

A total of 20 participants (mean age of 21.2 ± 2.5 years, 12 females) participated in the study. In each session (PRN, post recovery nap; RW, rested wakefulness; SD, sleep deprivation), participants completed a 5-minute resting state sequence (fixation cross) (Fig 1A). Participants also completed 3 cognitive tasks (Fig 1B). Two were focused on attention—the attention network task (ANT [22], 13 minutes) and the Mackworth clock task (MCT [23], 5 minutes). The other focused on working memory—the N-back task [24] (8 minutes). As expected, performance outcomes (accuracy and reaction time) across all tasks were significantly impaired following SD and improved following the recovery nap (S1 Text, Fig A in S1 Text, Table A in S1 Text).

Through EEG confirmation, 13 participants (65%) had brief sleep episodes (<10 seconds), and 4 (20%) fell asleep (>30 seconds) during the resting state sequences in the SD condition. In addition to excessive head motion (average framewise displacement: 0.16 ± 0.09mm in RW versus 0.22 ± 0.14mm in SD), this resulted in these sequences not being used in the main analyses, however results from the resting state data can be found in (SI Results in S1 Text, Fig B in S1 Text). None of the subjects in the final sample fell asleep during the cognitive tasks. Additionally, head movement inside the scanner during the cognitive tasks was not significantly different between the sessions (average framewise displacement across 3 tasks: 0.15 ± 0.08 mm (RW) versus 0.17 ± 0.09 mm (SD); F = 1.06, *p* = 0.301). Therefore, we focused our main analyses on task fMRI data during the RW, SD, and PRN states, as well as during the recovery nap consisting of NREM sleep.

The fMRI data across the multiple tasks were concatenated temporally for every subject, resulting in a 26-minute–long time series of blood oxygen level dependent (BOLD) signal (Fig 1C). The general task-specific activity (i.e., for all trials for the ANT and all blocks for the MCT and N-back tasks) was regressed out from the BOLD time series as has been implemented in previous studies [25,26] to reduce the influence of task-evoked activations in connectivity and integration analyses. The BOLD data sampled along the cortical surface were clustered into 400 parcels [27]. Integration was calculated at different levels in a constructed hierarchical model of the cortex. The whole cortex was divided into 7 networks, and each of these 7 networks were further divided into smaller subnetworks (17 networks in total), based on a widely used functional template of cortical networks [28]. We further divided the 17 subnetworks into smaller clusters (57 clusters in total, estimated through hierarchical clustering within each of the 17 subnetworks, Fig 1D, Fig C in S1 Text) to assess integration changes at an even more localised level than 17 networks. Total integration (*I*_*Tot*_) equates to the sum of within and between subsystem integration (i.e., within and between 7 networks at the whole cortex level). Differences in total integration were compared between the 3 vigilance states and NREM sleep within a Bayesian framework as has been used previously [20,21].

### Altered functional integration in the cerebral cortex during the sleep-deprived state

**Fig 2:**
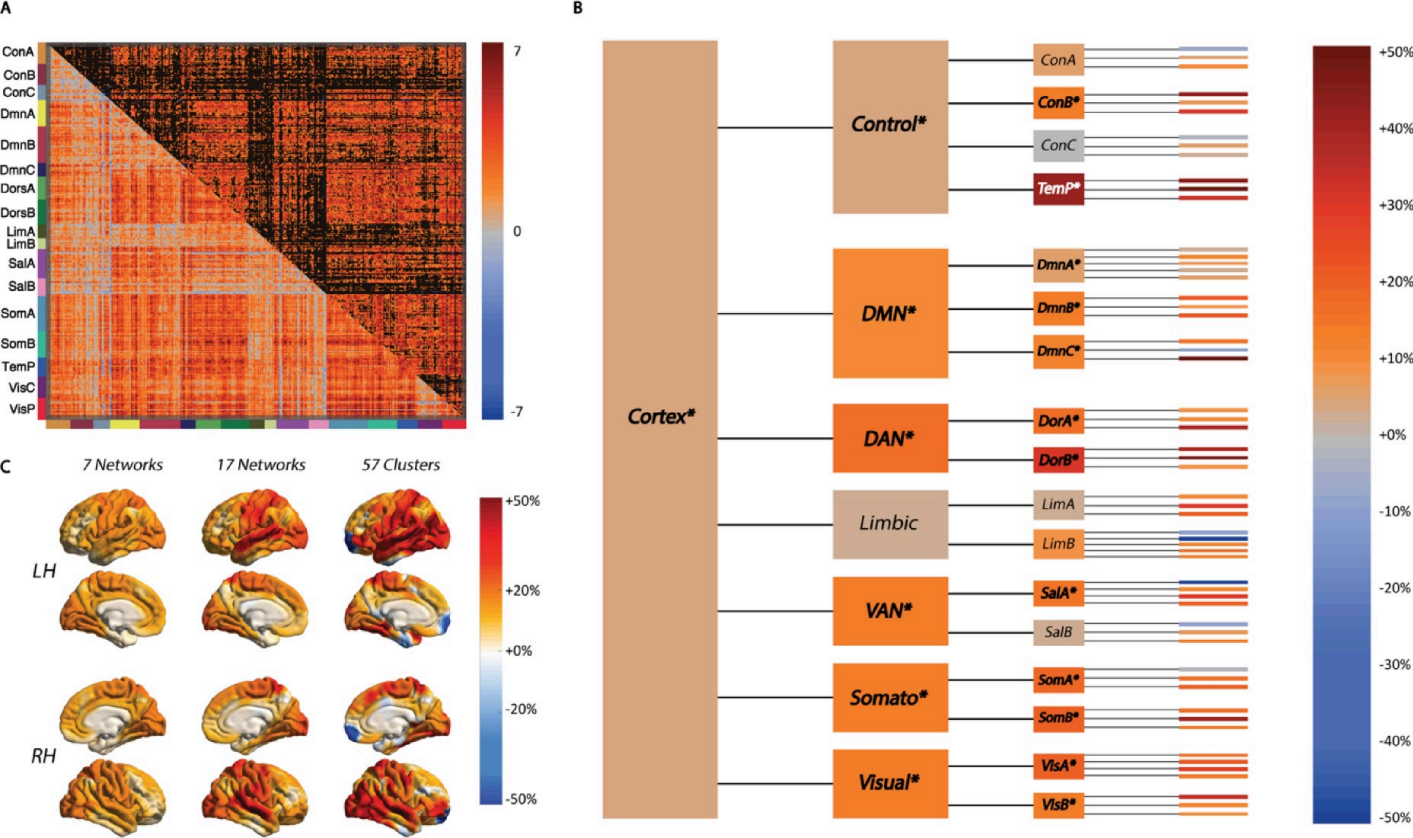
Changes in functional integration between large-scale cortical networks and regional clusters in the sleep-deprived state. **(A)** The *t* test matrix of change in functional correlations between the time series of 400 cortical parcels (during task performance) depicts a widespread increase in correlations between SD and WR states (unthresholded, lower triangle; thresholded P_FDR_<0.05, upper triangle). **(B)** Changes in integration (as a percentage of the value in the WR condition) are shown across different levels of a hierarchical model of the cortex: whole cortex, 7 networks, 17 networks, and 57 clusters. The total integration within the cortex increased from the WR to the SD state, but not within all networks (*italics depict significant changes measured through a Bayesian framework). **(C)** The % change in total integration at each level of the hierarchy mapped onto the cortical surface illustrates that the increase in integration is most focused towards the centre of the lateral surface. Data underlying this figure can be found in S1 Data. DAN, dorsal attention network; DMN, default mode network; PFDR, positive false discovery rate; Somato, somatomotor network; VAN, ventral attention (salience) network; WR, well rested.

Overall, there was a widespread increase in correlation values (functional connectivity) between cortical parcels from RW to SD (41% of edges significantly increased, Fig 2A). This coincided with an increase in the (*I*_*Tot*_) measured across the entire cortex. Fig 2B illustrates the changes in functional integration across distinct levels in the hierarchical model of the cortex. At the level of 7 cortical networks, there was an increase in integration within 6 of the 7 networks, with the exception of the limbic network (Table B in S1 Text). At the 17-network level, 12 networks demonstrated increased integration, while 1 of the ventral attention (B) and 2 of the frontoparietal control (A and C) and limbic networks showed no change (Table C in S1 Text). At the cluster level, there was more of a variety of change in integration, and 2 clusters within the limbic network demonstrated a substantial decreased integration from RW to SD. This pattern of change indicates that while increases in total integration were detectable when measured across the entire cortex, this was not uniform and preferentially affected certain networks and regions at lower, more localised, levels of the hierarchy (Fig 2C).

Further deconstructing these changes in integration following SD, we additionally calculated the degree of functional clustering within the cortex. Functional clustering estimates how integration is hierarchically organised within and between the constituent parts of a system, such as networks in the cortex. Specifically, the functional clustering ratio (FCR) is the ratio of within-network integration to between-network integration and quantifies the degree of functional segregation of a given system into subsystems [20]. There was a significant increase in the FCR from RW to SD at both the level of the entire cortex and within 4 of the 7 cortical networks: control, default mode, somatomotor, and ventral attention (Table D in S1 Text). The increase in both *I*_*Tot*_ and FCR was highly correlated across subjects (r = 0.92, *p* < 0.001). This indicates that the increase in *I*_*Tot*_ following SD was driven by integration within each network than any increased integration between networks (Fig D in S1 Text). After parsing each of the 17 networks into assemblies (clusters) based upon a hierarchical clustering of each network (Fig C in S1 Text), the only significant increases in the FCR were observable within the control (A), default mode (C), somatomotor (B), and ventral attention (A) networks (Table E in S1 Text).

To demonstrate the robustness of these findings to the choice of preprocessing or parcellation, we replicated these analyses without regressing out the task-specific activity (Table F in S1 Text) or when using network communities defined by a data-driven clustering of the functional connectome (via the Louvain algorithm [29]) rather than an a priori enforced template (Table G in S1 Text, Fig E in S1 Text). These analyses support the main findings. However, more cortical networks demonstrated significant increases in integration induced by SD when the task-evoked activity were not regressed out (7 of the Yeo7 networks and 15 of the Yeo17 networks), suggesting that task activations may interact with SD to further drive an increase in cortical within-network integration.

### Altered functional integration in the cerebral cortex is related to changes in cognitive performance

**Fig 3:**
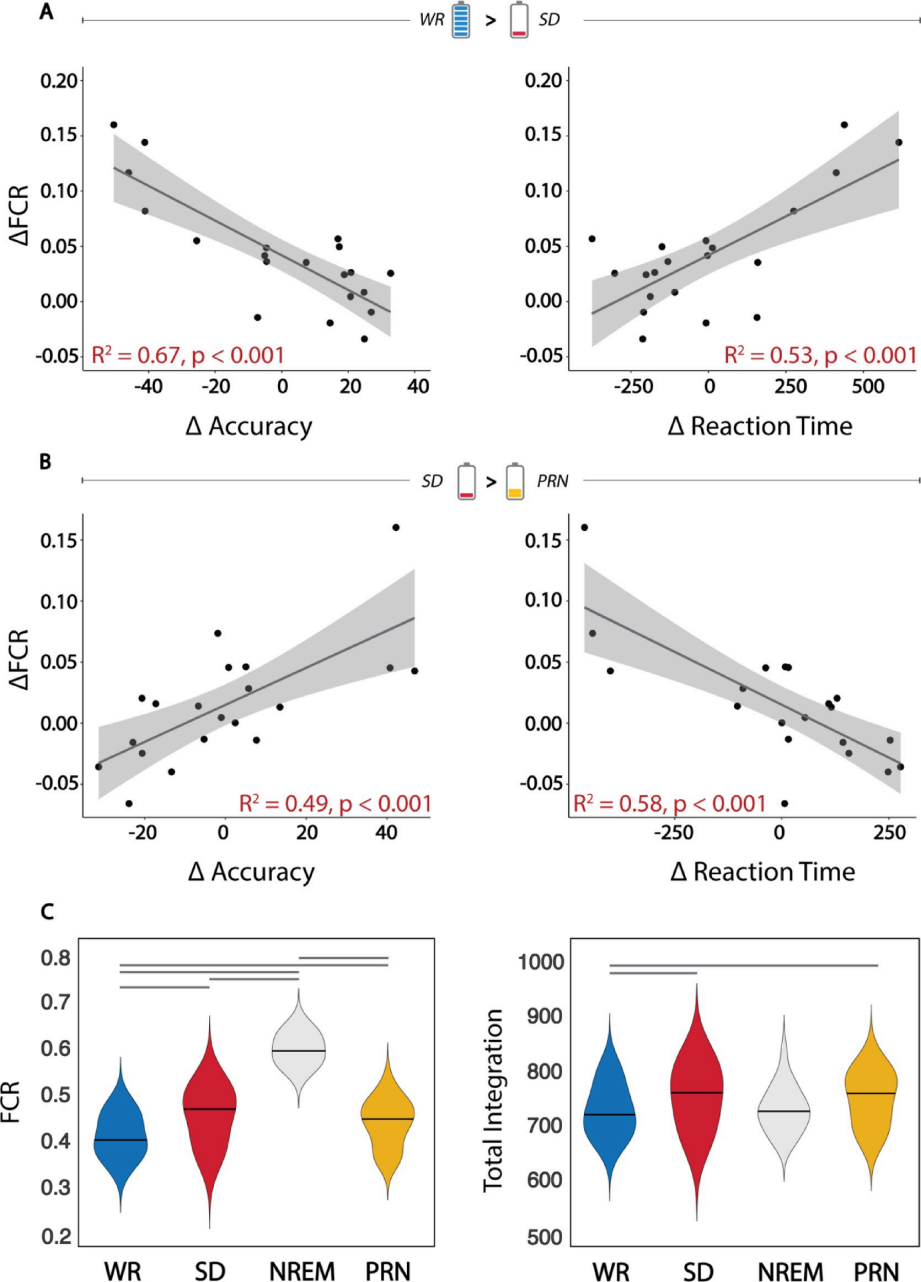
The changes in total integration and FCR is related to performance decline and recovery following SD. **(A)** There is a strong relationship between the change in total integration across the cortex and decrement in both performance accuracy and speed across 3 cognitive tasks from the WR to the SD state (top panel; performance on the x-axis is depicted as the first component from a principal component analysis on task outcomes). **(B)** The paired change in integration and performance was also observed for recovery after the nap. **(C)** The FCR increased from RW to SD, however, significantly increased in all subjects during the nap containing NREM sleep, indicating the role of FCR in conscious states (lines represent significant within-subjects differences between sessions). **(D)** Conversely, total integration increased within subjects from RW to SD, but there was a drop during the recovery nap despite remaining high upon waking after the nap (lines represent significant within-subjects differences between sessions). Data underlying this figure can be found in S2 Data. FCR, functional clustering ratio; NREM, nonrapid eye movement; PRN, post recovery nap; SD, sleep deprivation; WR, well rested.

To assess how the changes in brain activity patterns related to changes in cognitive performance, we first assessed a combination of performance change across all tasks. The difference in performance outcomes (SD–RW) on each of the 3 separate cognitive tasks were grouped into either accuracy (% of correct responses) or speed (reaction time, ms). These change scores for accuracy or speed were entered into 2 separate principal component analyses (PCAs) to obtain resulting the principal components that explained the most variance in accuracy or speed across the 3 tasks. The first component for accuracy (explaining 71% of the variance across tasks) was robustly and significantly negatively associated with the change in FCR within the cortex (Fig 3A). The first component for speed (explaining 95% of the variance) was also significantly positively correlated with the change in FCR across the entire cortex. Very similar results were observed for associations between the increase in (*I*_*Tot*_) and decrease in performance (SI Results in S1 Text, Fig F in S1 Text). The change in integration within each of the 7 networks was negatively correlated with change in accuracy and speed from RW to SD (SI Results in S1 Text). We also assessed the change in performance on each task separately in comparison to the change in (*I*_*Tot*_) during that task only. A greater increase in (*I*_*Tot*_) was significantly related to worse accuracy and speed in the ANT and Nback tasks, but not the MCT (Fig G in S1 Text).

### Changes in functional integration and cognitive performance following a recovery nap

A decrease in connectivity was observed from the SD to the PRN state, although to a lesser extent than the increase observed between RW and SD (16.7% of connections significantly decreased, Fig H in S1 Text). There was a small decrease in FCR across the entire cortex (Fig 3B). This was not correlated with the duration of the nap or the amount of slow wave sleep across individuals (SI Results in S1 Text). However, the subjects who had the greatest decrease in FCR from SD to the PRN showed the largest improvement in performance for both the accuracy and speed outcomes (Fig 3A). This recovery effect demonstrates a bidirectional link between changes in functional integration and changes in cognitive performance due to SD.

### Functional clustering of brain activity is robustly associated with conscious states

Following the SD condition, 19 of the 20 participants managed to successfully sleep inside the scanner, for an average of 50 ± 12 minutes (a sleep summary can be found in Table H in S1 Text). To ensure the reliability of integration measures in all conditions (RW, SD, PRN, and sleep), a stable period of 26 minutes (i.e., the same length as the concatenated task time series) consisting of NREM sleep (Stages N2 and N3) was selected from the fMRI scan sequence, and the same connectivity and integration metrics were calculated. During this recovery nap of NREM sleep, the FCR was significantly greater during NREM sleep than the wake states, for all subjects (Fig 3B). This was despite the amount of (*I*_*Tot*_) being comparable to the other wake states (Fig 3C). The increase in FCR and stability of (*I*_*Tot*_) was driven by both an increase in within-networks integration as well as a decrease in the integration between networks during the nap (SI Results in S1 Text, Fig D in S1 Text). These changes demonstrate that the altered balance of within-networks and between-networks integration is a characteristic feature of online versus offline brain states.

### Functional integration is linked to the amplitude of local and global signal fluctuations

**Fig 4:**
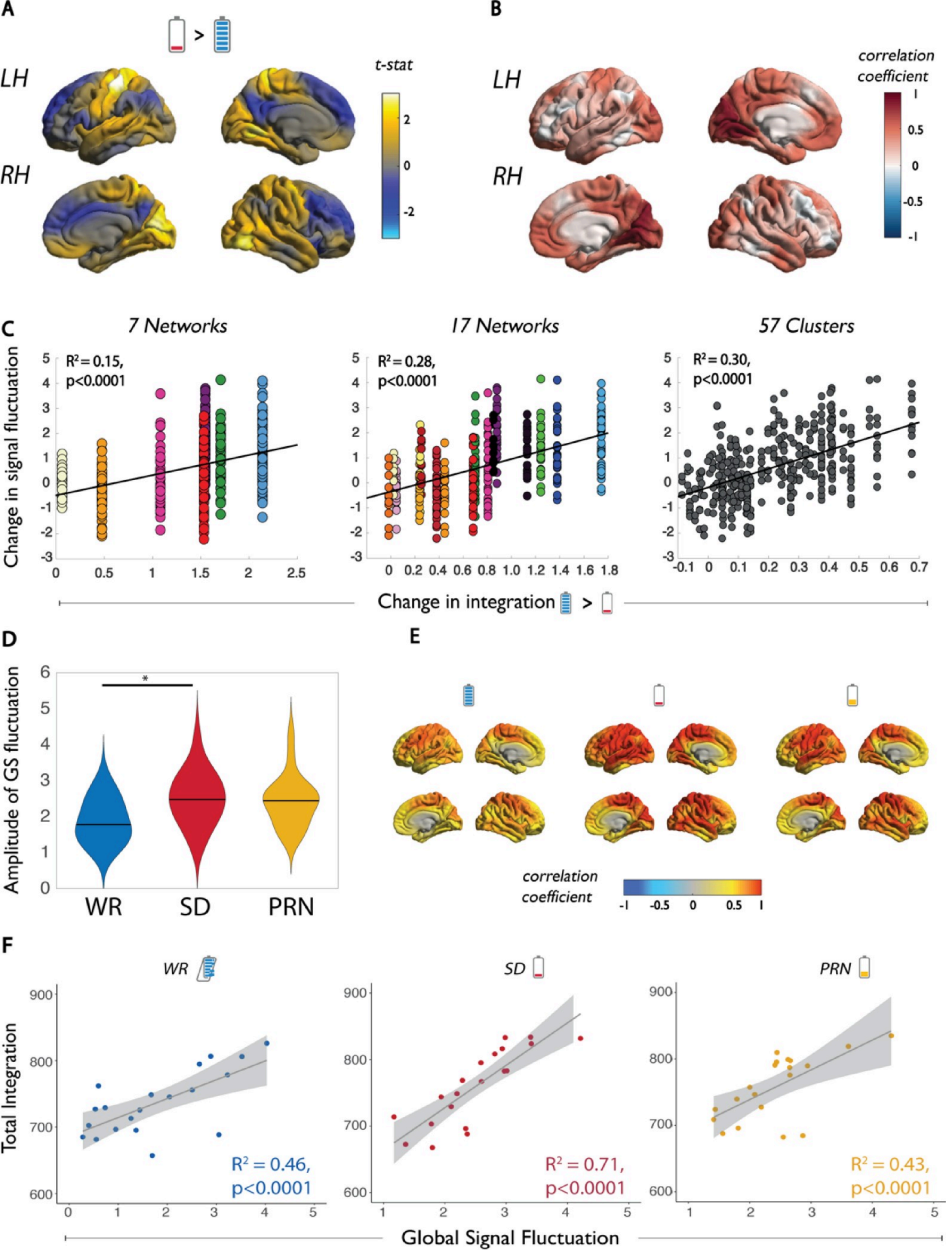
The relationship between functional integration and local and GS fluctuations. **(A)** The group average change in amplitude of signal fluctuation (standard deviation of signal) in each of 400 cortical parcels between SD and WR states. **(B)** The individual-level correlations between change in amplitude of the signal fluctuation and integration within 17 networks. **(C)** Correspondence between the group average change in amplitude of signal fluctuation with group average total integration within 7 networks, 17 networks, and 57 clusters (significance corrected with 100 spin permutations). **(D)** The distribution across subjects of the amplitude of the GS fluctuation in the WR, SD, and PRN states. **(E)** Group average correlation coefficient between each parcel’s time series and the GS in each state. **(F)** Individual relationships between total integration across the cortex and the amplitude of the GS fluctuation in the WR, SD, and PRN states. Data underlying this figure can be found in S3 Data. GS, global signal; PRN, post recovery nap; SD, sleep deprivation; WR, well-rested.

We investigated whether the increase in functional integration may be due to regional changes in the amplitude of brain activity fluctuations. Between the RW and SD states, the parcel-wise change in signal fluctuation amplitude (standard deviation of task-regressed signal in each 400 parcels) was greatest over the somatosensory cortex and peripheral visual cortex (Fig 4A). Across individuals, these changes in signal fluctuation amplitude were only correlated with individual-level changes in integration within the visual cortex (Fig 4B). However, the spatial correspondence between the group-level changes in signal fluctuation and changes in integration at the group level were all significantly correlated, and the relationship was strongest for integration changes at the cluster (assembly) level (Fig 4C).

In line with previous reports [5,15,16], the amplitude of the global signal fluctuation (standard deviation of the global signal time series) significantly increased from the RW to the SD state (Fig 4D). At the RW state, the time series of each parcel were only moderately associated with the global signal time series; however, this increased significantly across a widespread number of parcels, including somatomotor, visual, temporal, and parietal regions of the cortex during the SD state and persisted in the PRN state (Fig 4E). In each state, the (*I*_*Tot*_) was significantly correlated with the global signal fluctuation; however, the relationship was the strongest during the SD state (Fig 4F). This suggests that the global signal is strongly associated with measures of functional integration, but these measures become more tightly coupled in periods of reduced arousal, such as following SD. To ensure that these results were not driven by motion, the relationship between the global signal fluctuation and (*I*_*Tot*_) remained significant when average framewise displacement in each state was included as a covariate in the regression model (SI Results in S1 Text).

### Relationships with sleepiness and physiological markers of sleep pressure

Given the increase in both (*I*_*Tot*_) and the FCR within the cortex following SD, we tested whether these were related to the level of homeostatic sleep pressure. Firstly, the change in (*I*_*Tot*_) was not associated with subjective sleepiness (Karolinska sleepiness score [30]). Neither did the magnitude of the increase in (*I*_*Tot*_) or FCR following SD correlate with sleep latency, latency to Stage 3 NREM sleep, or the amount of slow wave activity (SWA) during the recovery nap (SI Results in S1 Text). Additionally, despite a slight slowing of EEG frequencies from RW to SD (1 to 4 Hz, SI Results in S1 Text), these changes were not related to the change in FCR or (*I*_*Tot*_). These all have been previously shown to be markers of homeostatic sleep pressure, suggesting that functional integration in the cortex is not merely a marker of enhanced sleep pressure. Furthermore, none of the aforementioned measures of homeostatic sleep pressure were related to the change in accuracy or reaction time from RW to SD (SI Results in S1 Text).

### Changes in subcortical connectivity

**Fig 5:**
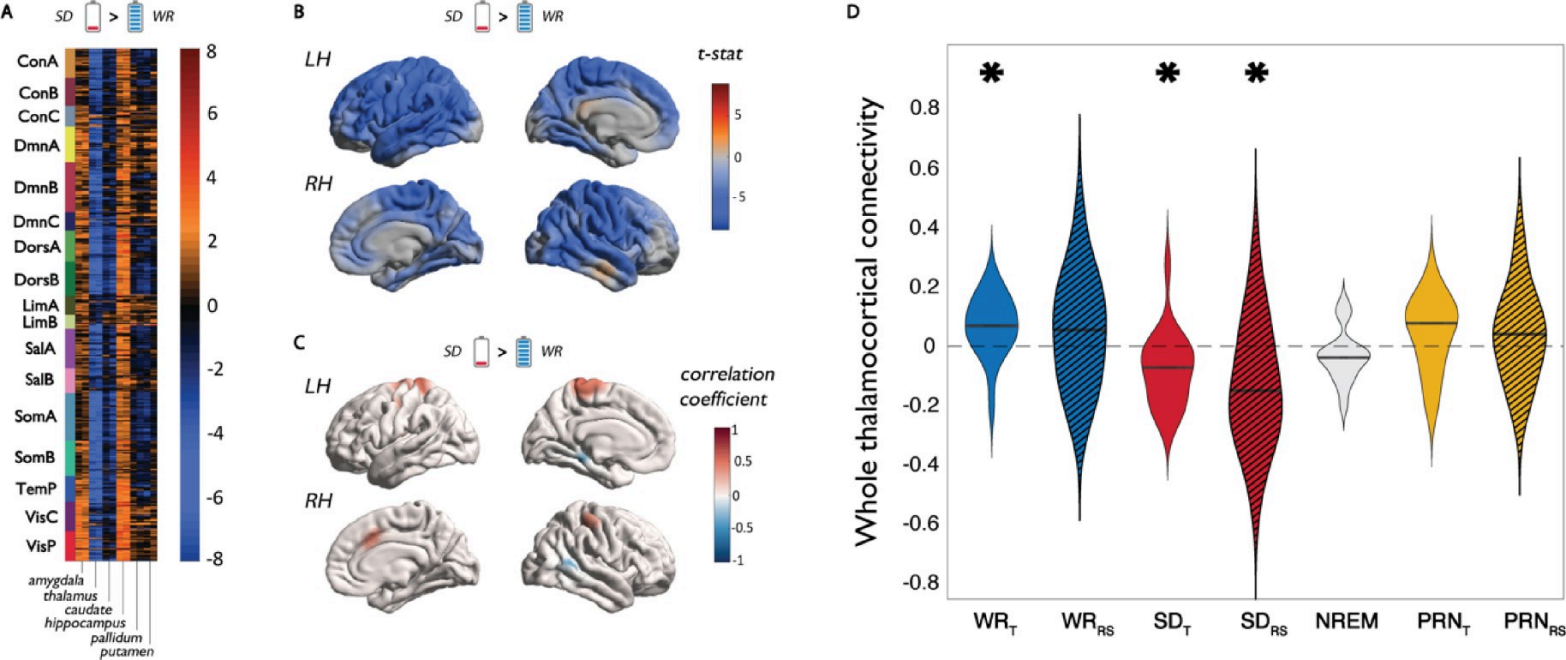
Changes in thalamocortical connectivity and the relationship to functional integration. **(A)** The group average change in connectivity (correlation of time series) between all 400 cortical parcels and 6 subcortical regions from SD and WR states during task performance. **(B)** Spatial map of changes in thalamocortical connectivity between SD and WR states during task performance. **(C)** The individual-level relationship between change in thalamocortical connectivity and integration within 17 networks during task performance. **(D)** The individual distributions of thalamocortical connectivity (averaged across the entire cortex) in the WR, SD, and PRN states. * Denotes average connectivity is significantly different from zero. Data underlying this figure can be found in S4 Data. NREM, nonrapid eye movement; PRN, post recovery nap; RS, resting state condition; SD, sleep deprivation; T, task condition; WR, well rested.

We also measured subcortical–cortical connectivity to investigate whether these interactions may influence changes in cortical integration in periods of reduced arousal. Out of 5 subcortical regions (amygdala, caudate, pallidum, putamen, and thalamus) and the hippocampus, there was a notable significant decrease in the correlations between the thalamus and widespread cortical regions following SD (Fig 5A). Connectivity changes with the thalamus were greatest in the somatomotor, dorsal attention, and temporal parietal networks (Fig 5B). However, no correlations between the change in thalamocortical connectivity and cortical integration were observed across individuals (Fig 5C). Neither were there any relationships observed between thalamocortical connectivity and cognitive performance or markers of sleep pressure (SI Results in S1 Text). Nevertheless, thalamocortical connectivity was lowest during the SD state, even less than during NREM sleep (Fig 5D). This was particularly accentuated in the resting state sequence following SD, a period of sleep–wake instability when the majority of subjects had sleep intrusions (Fig 5D).

## Discussion

These findings demonstrate that in states of low arousal (i.e., SD), the integration within cortical networks increases relative to integration between networks. These changes were observed during the performance of cognitive tasks, and, importantly, were strongly related to overall performance. The balance of these 2 integration terms, i.e., the FCR, can be viewed as a measure of the degree of functional segregation of a system into its constituent parts [11,20]. In other words, this signifies how much information cortical networks generate independently, compared with the information generated by the cortex as a whole. The FCR was found to increase across the cortex in the SD state and preferentially within certain functional networks (each divided, in turn, into smaller networks; Fig 2B), suggesting that these modifications in information integration were present when measured across different hierarchical levels of the cortex.

We detected the greatest FCR during the NREM recovery nap (Fig 3C), consistent with previous observations during NREM sleep [20,31,32]. Although previous comparisons of NREM sleep were with the wakeful resting state, the current findings demonstrate that these effects generalise to other states of wakefulness (i.e., task performance). The increase in FCR was driven by both an increase in within-network integration as well as a significant decrease in between-network integration. It is widely accepted that sleep is not merely a state of quiescence, but an active brain state of self-organised, endogenous activity mostly cut off from the outside world. The level of consciousness has been hypothesised to be related to the degree of integrated information in the brain, that is, the information generated by the interactions in a whole system, beyond the information generated by the parts [33,34]. This is consistent with our observations of segregated brain activity during the recovery nap. Alternatively, in the SD state, the integration between networks remained the same as the RW state, which is coherent with the fact that subjects remained in a conscious, wakeful state. However, given the increase of within-network integration, the FCR was still significantly higher when compared to RW. In summary, these results suggest that following prolonged wakefulness, the proportional integration and segregation of brain activity within structured cortical networks appears to be driven from levels in well RW towards those observed during offline states, such as NREM sleep, suggesting that there exists an underlying continuum of functional segregation from RW to NREM sleep. We were not able to robustly measure the differences between NREM2 and NREM3 in this study with the same amount of consecutive data points as in the wake conditions; however, other studies suggest that brain networks are even more segregated during NREM3 compared to NREM2 sleep [35] and that total integration decreases in proportion to SWA [20].

We have recently shown that the primary axes of differentiation in functional connectivity (i.e., functional gradients) across the cortex do not undergo major changes following SD [36]. This is consistent with the current findings, although within-network integration increased, and between-network functional connectivity remained stable (Fig D in S1 Text). Furthermore, the gradient approach is mainly sensitive to the shape of the functional connectivity distributions rather than variations in amplitude. Indeed, in this study, we found that changes in the integration of brain activity are related to elevated amplitude of brain signal fluctuations, particularly the global signal (Fig 4F). We were able to confirm that this relationship was not due to increased motion during the SD state (SI Results in S1 Text). The reason behind an increase in signal amplitude during SD is not clear. However, one emerging candidate for a primary role of sleep is the flushing of metabolic waste through the mixing of cerebrospinal and interstitial fluid via the glymphatic system [37,38]. Large oscillations of fluid inflow into the perivascular space occur during sleep and are tightly coupled with large amplitude EEG slow waves [39,40]. Thus, a buildup of waste products from extended periods of cellular activity in the brain may trigger the mechanisms (i.e., large amplitude slow waves) for the glymphatic system to begin clearing waste, even in the wake state [38]. Additionally, it is also plausible that a change in respiration or cardiac activity due to parasympathetic drive could account for changes in BOLD activity amplitude, as cerebral blood flow increases after SD [41], and the greatest changes in integration were detected nearest to the middle cerebral artery [42]. Finally, given the assumptions of our model of integration, in particular that the BOLD data at each time point are temporally independent and identically distributed (i.i.d) realisations of a 400-dimensional random variable y [21], it is possible that potential sample dependencies across time could contribute to an overestimation of integration measures within the data. This could mean that the increased integration we observed in the SD condition is due to an increase in temporal dependencies in the data. This should be investigated in future studies investigating the dynamics of brain activity in the SD state. The effect of spatial dependence in the BOLD time series would have had a negligible impact on these findings, as it has been shown that this effect is important at the small spatial scale (i.e., few voxels) and is less important at the regional level (i.e., >30 mm), the latter of which was implemented in this study [43].

Our results demonstrate that disruption to the balance between integration and segregation of cortical networks has a significant impact on effective and efficient cognitive performance (Fig 3A), which was shown to be specific to executive attention and working memory rather than vigilance (Fig G in S1 Text). This effect was bidirectional, as both increased FCR following SD negatively correlated with performance change, while the decrease in FCR following a recovery nap was associated with the greatest cognitive improvement. Maintaining a balance between integration and segregation of information flow is thought to be crucial for distributed brain networks to execute effective cognitive function [7,11]. In particular, the ability to dynamically fluctuate between integrated and segregated brain states may be the primary mechanism that supports ongoing cognitive processes [44,45]. Information generated by the brain would theoretically decrease if dynamic states become more homogenous (indicated by an increase in total and within-network integration or global signal amplitude). Evidence from resting state fMRI studies have demonstrated that SD results in increased time spent in dynamic states associated with low vigilance [46–48]. Also, it has recently been shown that brain connectivity integration changes during resting state are coupled with arousal fluctuations [49]. However, how SD impacts state dynamics during the performance of cognitive tasks remains to be seen.

We also replicated previous findings demonstrating that SD results in disruptive changes of thalamocortical functional connectivity, including the introduction of negative correlations [5,50]. The underlying mechanisms and physiological meaning of negative functional connectivity are still unclear [51–53]. However, the disruption of thalamocortical connectivity would have a significant impact on the maintenance of cortical arousal. The intralaminar and midline nuclei of the thalamus, with strong inputs from brainstem nuclei, are considered as part of the ascending reticular activating system that stimulate the cortex and facilitate the generation of intrinsic functional modes [17,54,55]. The thalamus also serves as the major hub for gating the flow of information into the cerebral cortex by blocking incoming signals through synaptic inhibition of thalamocortical relays. This is proposed to be the main mechanism contributing to shifting the brain from an aroused state into an “offline” state such as sleep [56].

However, we observed no relationship between the change in thalamocortical connectivity and cortical integration or cognitive performance across subjects (Fig 5C). Specifically, almost every subject expressed decreased (or anticorrelated) thalamocortical connectivity in the SD state, while there were more varied changes in integration between the RW and SD states across subjects (some not exhibiting relative increases in integration or FCR after 24 hours of SD). There are 2 possible explanations for these findings. Firstly, during prolonged wakefulness, thalamocortical disconnection may precede changes in cortical integration, the latter occurring after different durations of SD across individuals. This is supported by evidence that during sleep onset, thalamic deactivation (i.e., thalamic activity decreasing to overall sleep levels) precedes cortical changes by several minutes [57,58]. This would also be consistent with the observation that the thalamocortical connectivity was at its lowest in the SD resting state sequence (Fig 5D), when it was confirmed through EEG that the majority of subjects experienced brief entry into sleep. An alternative possibility is that disrupted thalamocortical connectivity is a state-based effect of SD, while altered cortical integration underlies interindividual differences in cognitive performance (or cortical “reserves”) following SD. To compare these hypotheses would require repeated measurements of brain activity across an extended period of prolonged wakefulness. Regardless, these results further elucidate the relationship between the thalamus and cortex, demonstrating that, in some individuals, the cortex can still maintain an optimal balance of integration and segregation required for cognitive processing despite becoming functionally disconnected from the thalamus. As has been proposed, the ascending modulatory transmitter systems mostly provide the necessary arousal to tune the state and excitability of the different parts of the cortex, which allow for the appropriate analysis of sensory information (i.e., cognitive processing and behavioural responses) [56]. Therefore, thalamocortical disruption following SD may not directly cause impaired cognition, but rather be a precipitating factor via altered functional integration in the cortex.

## Conclusions

In conclusion, SD appears to impact the balance of integration and segregation of brain activity. Whether these changes are completely neurogenic or arise from systemic processes involved in maintaining homeostasis of the cellular environment in the brain remain to be elucidated. However, they appear independent from conventional markers of homeostatic sleep pressure and changes in thalamocortical connectivity. Regardless, the disruption of information integration in the brain is significantly linked to the extent of cognitive impairment experienced by individuals following SD. Future perspectives should focus on why integration and segregation of brain activity is impaired following SD and how this impacts the dynamics of integrated network states during cognitive task performance.

## Materials and methods

### Population and experimental design

A total of 20 participants were recruited using advertisements posted online and within Concordia University, Montreal. This study was approved by the Comité central d’éthique de la recherche (CCER), established by the Ministère de la Santé et des Services sociaux in Quebec. This ethical review of research adheres to the principles expressed in the Declaration of Helsinki. Informed written consent was obtained from all participants. All participants were 18 to 30 years old, healthy, and regular sleepers, and none were taking any medication. Participants made 3 visits to the sleep laboratory, including a night for habituation and screening to rule out sleep disorders, a normal night (8-hour sleep, RW) and experimental night (0-hour sleep, SD). Each morning in the RW and SD conditions, participants completed one resting state sequence and 3 cognitive tasks: the ANT, MCT, and the N-back task. In the SD condition, participants were provided a 60-minute opportunity to sleep inside the MRI, and the tasks and then the resting state sequence were repeated. The order of the RW and SD sessions was counterbalanced across subjects (SI Materials and methods in S1 Text).

### Analysis

The change in performance outcomes on each of the 3 separate cognitive tasks were grouped into either accuracy (% of correct responses) or speed (reaction time, in ms). This provided 2 data matrices of size #subjects × #tasks, one matrix containing accuracy scores and one containing reaction times. The change scores for accuracy or speed were entered into separate PCAs, in order to provide a global estimate of performance. The first principal component from each PCA was extracted and used as a single measure for accuracy or speed across the 3 tasks.

### fMRI data acquisition and analysis

MRI scanning was acquired with a 3T GE scanner (General Electric Medical Systems, Wisconsin, United States) using an 8-channel head coil. Structural T1-weighted images with a 3D BRAVO sequence, and functional echo planar images were acquired. Data were preprocessed using the fMRIPrep [59] and xcpEngine [60] toolboxes (for detailed steps, see SI Materials and methods in S1 Text). The preprocessed BOLD time series for each subject was projected onto the cortical surface and smoothed along the surface using a 6-mm smoothing kernel using the FreeSurfer software package. For each subject and session, the general task-specific activity (convolved with a hemodynamic response function) was regressed out from the BOLD time series, and the time series from all tasks were concatenated into one time series per subject and condition.

### Network and assembly identification

For all analyses of task data, the BOLD time series for each vertex on the fsaverage surface was assigned to one of 400 cortical parcels from a predetermined standardised template of functionally similar cortical regions [27]. The time series for all the vertices corresponding to each parcel were averaged to give one mean time series per parcel, resulting in a total of 400 time series across both cortical hemispheres. We then divided the cortex in 3 separate steps, which allowed us to arrange the data in a hierarchical framework comprising 4 levels (Fig B in S1 Text). Firstly, at the cortex level, each parcel was assigned to either 1 of 7 functional networks, taken from a template based on a large sample of resting state data [28]. At the second level of the hierarchy (7 networks), each parcel was assigned to 1 of 17 functional networks taken from a higher resolution version of the same template [28], and these 17 networks were clustered into 1 of the 7 original networks based on naming convention. At the third level (17 networks), each of the 17 functional networks were further partitioned into assemblies (clusters) based upon a hierarchical clustering technique, resulting in a partitioned composed of 57 clusters. To achieve this last level of the hierarchy, the averaged correlation matrix was computed for each network during the RW session and thresholded at *p* < 0.05. The thresholded correlation structure was computed for each of the 17 networks, and their structure was assessed by a hierarchical clustering that maximised intraclass similarity, defined as $\sqrt{\left(1-r^{2}\right)}$, where r is the correlation coefficient between 2 regions. By thresholding the similarity trees at the level of the highest increase of intracluster distance, the 17 networks were divided into assemblies of areas (Fig E in S1 Text). Subcortical regions were extracted from the FreeSurfer segmentation of the FSL MNI152 template represented by 8 regions of interest (ROIs) corresponding to the left and right hemispherical thalamus, striatum, hippocampus, and amygdala. Partition into community structure and analyses of resting state data are described in SI Materials and methods in S1 Text.

### Functional connectivity and integration

The mean time course of each parcel or volume was correlated to the mean time courses of all other parcels, and the correlation matrices were z-scored. Significance was corrected at *p* < 0.05 using the false discovery rate method [61].

Functional integration was calculated as follows. The functional data were considered as *N* ROIs (parcels) characterised by their mean time courses *y* = (*y*_1_,…,*y*_*N*_) gathered into *K* systems (networks) defined as *S* = {*S*_1_,…,*S*_*K*_}. For any partition of *y*, integration (or mutual information) can then defined as the Kullback–Leibler information divergence between the joint distribution *p*(*y*_1_,…*y*_*K*_) and the product of the marginal distributions of the N-dimensional fMRI BOLD time series *y* divided into *K* subsets, such that

$$I[y_{1},\ldots ,y_{K}]=D_{KL}[p(y_{1},\ldots ,y_{K});\prod_{k=1}^{K}p(y_{k})],$$

which can be rewritten as

$$I[y_{1},\ldots ,y_{K}]=\sum_{k=1}^{K}H(p(y_{k}))-H(p(y_{1},\ldots ,y_{K})),$$

where *H* is the Shannon entropy measure [21]. For multivariate normal data with mean *mu* and covariance matrix *Sigma*, entropy can be computed as

$$H(p(y))=\frac{1}{2}ln(|Sigma|)$$

where |*Sigma*| refers to the determinant of the covariance matrix. As described in [21], the total integration (*I*_*Tot*_) can be decomposed, according the organisation of regions into systems, as the sum of each system’s integration relative to its regions (within-system integration) and a between-system integration term

$$I[y_{1},\ldots ,y_{N}]=I_{ws}+I_{bs}$$

or, alternatively,

$$I[y_{1},\ldots ,y_{N}]=\sum_{k=1}^{K}I({\left(y_{n}\right)}_{n\in S_{k}})+I[y_{S_{1}},\ldots ,y_{S_{K}}].$$

Integration was calculated at different levels in our proposed hierarchical model of the cortex. Firstly, integration was calculated across the whole cortex where *N* = 400 parcels were gathered into 7 networks. Secondly, for each of 7 networks separately, the N parcels were gathered into small subnetworks (Yeo 17 networks), where each of the 7 networks contained a minimum of 2 out of 17 subnetworks. Finally, for each of the 17 subnetworks, the N parcels were divided into K of the 57 clusters (assemblies), where each of the 17 networks contained a minimum of 2 out of 57 clusters.

We defined the FCR as the ratio of the integration within subsystems (*I*_*ws*_), compared to the integration present between these subsystems (*I*_*bs*_) [20]. It is a measure of clustering inside a given system because an increase in FCR indicates that subsystems become proportionally more independent of each other. In other words, it quantifies the degree of functional segregation of a given system into subsystems:

$$FCR=I_{ws}/I_{bs}.$$

The FCR was also computed for each network or cluster at each level of the hierarchy.

### EEG data acquisition and analysis

EEG was acquired using an MR compatible 256 high-density geodesic sensor EEG array (Electrical Geodesics (Eugene, OR, USA), Magstim EGI). EEG data were recorded at 1,000 Hz referenced to Cz using a battery-powered MR-compatible 256-channel amplifier. Electrocardiography (ECG) was also collected via 2 MR compatible electrodes through a bipolar amplifier (Physiobox (Eugene, OR, USA), Magtism EGI). The EEG data were corrected for MR gradient ballistocardiographic pulse-related artefacts using the Brainvision Analyzer (Brain Products, Gilching, Germany). The MR-denoised EEG signal was band-pass filtered between 1 and 20 Hz to remove low-frequency drift and high-frequency noise, down-sampled to 250 Hz, and re-referenced to the linked mastoids. Scoring of the sleep session was performed in conjunction by 2 trained scorers (NEC and AAP) using the Wonambi toolbox (https://github.com/wonambi-python/wonambi) (using the channels Fz, F3, F4, C3, C4, O1, and O2) in order to obtain measures of sleep including total sleep time and the duration of sleep stages. For the analysis of wake EEG, eye blink artefacts were removed with independent component analysis (ICA) using the MNE Python package. The ICA was fit on each EEG time series concatenated across tasks for each subject and condition. The number of components was set to 15, with a random seed to ensure the uniformity of components. The resulting ICA components were then compared to electrooculogram (EOG) electrodes any independent components (ICs) that matched the EOG pattern were automatically marked for exclusion. Any matching components were then excluded from the original signal, and this new time series was then used for all subsequent analysis. Power spectral analysis was also computed using MNE, via the Welch method using a frequency resolution of 1 Hz.

### Statistical analysis

#### Bayesian sampling scheme

Probable values of integration and FCR were inferred from the data using a Bayesian numerical sampling scheme that approximates the posterior distribution of the parameters of interest [21]. We first ran Gibbs sampling on the model to propose a numerical approximation of p(Sigma|y). Using this sampling procedure, we obtained 1,000 samples from the posterior distribution of the group covariance matrix (*Sigma*s = 1,…,1,000) in either condition (RW, SD, and PRN). From each sample *Sigma*s, we computed the corresponding values of integration (Iws and Ibs) and FCR. For each measure and condition, we therefore obtained a frequency histogram that, by construction, approximated the posterior distribution of that measure given the data. The samples were then used to provide approximations of either statistics (e.g., mean and SD) or probabilities (e.g., probability of an increase between RW and SD) approximated as the frequency of that increase observed in the samples. This also allowed to approximate the posterior probability p(A|y) of the assertion, e.g., FCR_SD_ > FCR_RW_ using the equation

$$P(A|y)\approx \frac{1}{S}\#\{FCR_{SD\{s\}}>FCR_{RW\{s\}}\},$$

where # stands for the cardinal function of a set. For analyses between conditions, a probability of difference >0.95 was considered significant.

During the sampling procedure, a covariance matrix was also estimated for each individual. Thus, similarly to the group-level analysis, we compared the resulting estimates of FCR at the individual level with other individual metrics (e.g., performance measures, global signal fluctuation, and thalamocortical connectivity) using product–moment correlations.

#### Spatial correspondence between cortical maps

For the comparison between brain maps (Fig 3), we used a spatial permutation framework to generate null models of overlap [62]. Spatial permutation of brain maps was performed using 100 angular permutations of spherical projections of the cortical surface. This approach calculates a correspondence statistic between 2 maps while controlling for spatial autocorrelation in the data.

## Supporting information

S1 TextThis file contains Supporting information Materials and methods and Results that provide more detailed information regarding how the study was conducted and control analyses that provide more information surrounding the results reported in the main text.This file also contains Supporting information Tables A–H and Figs A–H. **Table A:** Mean task performance scores in each state. **Table B:** The *I*_*Tot*_ within 7 cortical networks, in the WR, SD, and PRN states. **Table C:** The *I*_*Tot*_ within 17 cortical networks, in the WR, SD, and PRN states. **Table D:** The FCR within 7 cortical networks, in the WR, SD, and PRN states. **Table E:** The FCR within 17 cortical networks, in the WR, SD, and PRN states. **Table F:** Changes in total integration and FCR across the whole cortex and cortical networks, when task-specific activity was not regressed out of the time series for each parcel. **Table G:** Changes in total integration and FCR across the whole cortex using networks defined by the Louvain modularity algorithm. **Table H:** Mean sleep statistics during the nap. **Fig A:** Performance outcomes (accuracy + reaction time) across all tasks were significantly impaired following SD and improved following the recovery nap. **Fig B: (A)** The matrix of change in resting state functional correlations between the time series of 100 cortical parcels depicts a widespread increase in functional connectivity from WR to SD states, however, to a lesser extent than during tasks (unthresholded, lower triangle; thresholded P_FDR_<0.05, upper triangle). **(B)** Changes in integration are shown across 2 levels of a hierarchical model of the cortex: whole cortex and 7 networks (the other levels could not be calculated with a 100 parcellation templat, due to spatial resolution and that integration calculations required >1 parcels per region). The total integration within the cortex increased from the WR to the SD state and within 6 out of the 7 functional networks (white italics depict significant changes, variations probability >0.95). **(C)** The change in total integration mapped onto the cortical surface illustrates a widespread increase in integration across the entire cortex during resting state. **Fig C:** Each of the 17 Yeo functional networks were partitioned into assemblies (clusters) based upon a hierarchical clustering that maximised intraclass similarity. A total whole brain partition of 57 clusters was resulting from this procedure. To achieve this, the averaged correlation matrix was computed for each network during the WR session and thresholded at *p* < 0.05. The thresholded correlation structure was computed for each of the 17 networks, and their structure was assessed by a hierarchical clustering that maximised intraclass similarity, defined as $\sqrt{\left(1-r^{2}\right)}$, where r is the correlation coefficient between 2 regions. By thresholding the similarity trees at the level of the highest increase of intracluster distance (dotted line), the 17 networks were divided into assemblies of areas. Clusters are displayed upon the cortical surface (upper right). **Fig D:** There was a significant increase in total integration from RW to SD. This increase was due to an increase in within-systems (networks) integration, with no change in integration between networks. Alternatively, while there was no change in total integration during the NREM nap, there was a significant increase in within-systems and decrease in between-systems integration (solid lines represent significant differences between states). **Fig E: (A)** Five networks (communities) were detected from the functional connectome in the WR condition using the Louvain modularity algorithm of the Brain Connectivity Toolbox. Visually, these appear to cover well described cortical networks in the literature: visual; somatomotor; default mode; limbic; attentional networks. **(B)** When hierarchical integration was computed across these extracted networks, there was an observed increase from WR to the SD condition across the level of the entire cortex and also within 4 of the 5 networks (*bold italics represent significant differences across states). **Fig F: (A)** Associations between the increase in total integration and cognitive performance. Similar to the FCR, there was a significant negative relationship between change in integration and accuracy and a positive relationship with speed performance from RW to SD. **(B)** There was also a significant negative relationship between change in integration following the PRN and the change in performance from SD to PRN. **Fig G:** The change in performance from RW to SD on each task separately in comparison to the change in integration of cortical BOLD activity during each task. A greater increase in total integration was significantly related to worse accuracy and speed in the ANT and Nback tasks, but not the MCT. **Fig H: (A)** Overall a decrease in functional connectivity was observed from the SD to the PRN state. **(B)** Changes in integration are shown across different levels of a hierarchical model of the cortex: whole cortex, 7 networks, 17 networks, and 57 clusters. The total integration within the cortex decreased minimally from the WR to the SD state and at the network level decreases were only observed within the default mode, somatomotor and visual networks (bold white italics depict significant changes, FDR corrected). ANT, attention network task; BOLD, blood oxygen level dependent; FCR, functional clustering ratio; MCT, Mackworth clock task; NREM, nonrapid eye movement; P_FDR_, positive false discovery rate; PRN, post recovery nap; RW, rested wakefulness; SD, sleep deprivation; WR, well rested.(DOCX)

S1 Data This file contains data underlying Fig 2. (ZIP)

S2 Data This file contains data underlying Fig 3. (ZIP)

S3 Data This file contains data underlying Fig 4. (ZIP)

S4 Data This file contains data underlying Fig 5. (ZIP)

## Abbreviations

ANT: attention network task
BOLD: blood oxygen level dependent
CCER: Comité central d’éthique de la recherche
ECG: electrocardiography
EEG: electroencephalography
EOG: electrooculogram
FCR: functional clustering ratio
fMRI: functional magnetic resonance imaging
ICA: independent component analysis
i.i.d: independent and identically distributed
MCT: Mackworth clock task
NREM: nonrapid eye movement
PCA: principal component analysis
PRN: post recovery nap
ROI: region of interest
RW: rested wakefulness
SD: sleep deprivation
SWA: slow wave activity
