# Supplementary material for: An altered balance of integrated and segregated brain activity is a marker of cognitive deficits following sleep deprivation
Source: PLoS Biol. 2021 Nov 4;19(11):e3001232. doi: 10.1371/journal.pbio.3001232 (PMC8568176; doi:10.1371/journal.pbio.3001232)
Supplement: S1 Data — (ZIP) [file pbio.3001232.s002.zip › S1_Data/Data_underlying_figure2.docx]

**Figure 2A** – The numerical t-statistic values underlying the lower triangle of the matrix are to be found in the sheet ‘Ttest’ in the file S1_Data.xlsx. The False Discovery Rate corrected p-values for these tests are to be found in the sheet ‘Pvalues_fdr’ in the file S1_Data.xlsx. The individual functional connectivity matrices for which this t-test was performed are found in the files fc_WR.mat and fc_SD.mat.

**Figure 2B** – The 1000 samples resulting from the Gibbs sampling of the functional covariance matrices for the whole cortex in each state (WR, SD and PRN) are to be found in the sheet ‘Integration_WC’ in the file S1_Data.xlsx. The 1000 samples for each of the N7, N17 and A57 networks and assemblies for each state are to be found in the sheets ‘Integration_N7’, ‘Integration_N17’, ‘Integration_A57’ respectively. The means, standard deviations and significance levels calculated from Bayesian inference scheme, for all networks and assemblies in each state, are to be found in the sheet ‘Integration_Stats’ in the file S1_Data.xlsx.

**Figure 2C** – The data for these cortical surface figures are same the t-test values for the N7, N17 and A57 networks and regions, which are to be found in the sheet ‘Integration_Stats’ in the file S1_Data.xlsx.
