# Supplementary material for: An altered balance of integrated and segregated brain activity is a marker of cognitive deficits following sleep deprivation
Source: PLoS Biol. 2021 Nov 4;19(11):e3001232. doi: 10.1371/journal.pbio.3001232 (PMC8568176; doi:10.1371/journal.pbio.3001232)
Supplement: S2 Data — (ZIP) [file pbio.3001232.s003.zip › S2_Data/Data_underlying_figure3.docx]

**Figure 3A** – The individual values for the Itot in each state (WR, SD, NREM, PRN) are to be found in the sheet ‘Itot_states’ in the file S2_Data.xlsx.

**Figure 3B** – The individual values for the FCR in each state (WR, SD, NREM, PRN) are to be found in the sheet ‘FCR_states’ in the file S2_Data.xlsx.

**Figure 3C** – The data underlying the scatterplots in figure 3C are to be found in the sheet ‘Figure3C_scatterplots’ in the file S2_Data.xlsx. The raw cognitive data can be found in the sheet ‘CognitiveData’. The FCR change values are calculated from the values in sheet ‘FCR_states’ in the file S2_Data.xlsx.
