# Supplementary material for: An altered balance of integrated and segregated brain activity is a marker of cognitive deficits following sleep deprivation
Source: PLoS Biol. 2021 Nov 4;19(11):e3001232. doi: 10.1371/journal.pbio.3001232 (PMC8568176; doi:10.1371/journal.pbio.3001232)
Supplement: S3 Data — (ZIP) [file pbio.3001232.s004.zip › S3_Data/Data_underlying_figure4.docx]

**Figure 4A** – The amplitude of the signal fluctuation (standard deviation of raw signal) for each of the 400 cortical parcels, for each state, is to be found in the sheet ‘Figure4A_raw’ in the file S3_Data.xlsx. The t-test values and p-values for each parcel are to be found in the sheet ‘Figure4A_ttest’.

**Figure 4B** – The mean change in total integration (7 network level) and the mean change in the amplitude of the signal fluctuation (change in the standard deviation of raw signal) for each of the 400 cortical parcels from the WR to the SD state, is to be found in the sheet ‘Figure4B_N7’ in the file S3_Data.xlsx. The data values corresponding to these changes at the 17 network level and 57 assemblies level, are to be found in the sheets ‘Figure4B_N17’ and ‘Figure4B_A57’ respectively.

**Figure 4C** – The individual changes in total integration (17 network level) and the individual changes in the amplitude of the signal fluctuation (change in the standard deviation of raw signal) for each of the 400 cortical parcels from the WR to the SD state, is to be found in the sheet ‘Figure4C_raw’ in the file S3_Data.xlsx. The correlations (r-coefficient and p-value) between these values for each of the 400 cortical parcels is to be found in the sheet ‘Figure4C_correlations’.

**Figure 4D** – The individual values for the amplitude of the global signal fluctuation (change in the standard deviation of the global signal) is to be found in the sheet ‘Figure4D_raw’ in the file S3_Data.xlsx.

**Figure 4E** – The correlations (r-coefficient and p-value) between the raw signal and the global signal for each of the 400 cortical parcels for each state (WR, SD and PRN) is to be found in the sheet ‘Figure4E_correlations’ in the file S3_Data.xlsx.

**Figure 4F** – The data underlying the scatterplots in Figure 4F are to be found in the sheet ‘Figure4F_scatterplots’ in the file S2_Data.xlsx.
