# Supplementary material for: An altered balance of integrated and segregated brain activity is a marker of cognitive deficits following sleep deprivation
Source: PLoS Biol. 2021 Nov 4;19(11):e3001232. doi: 10.1371/journal.pbio.3001232 (PMC8568176; doi:10.1371/journal.pbio.3001232)
Supplement: S4 Data — (ZIP) [file pbio.3001232.s005.zip › S4_Data/Data_underlying_figure5.docx]

**Figure 5A** – The numerical t-statistic values underlying the matrix are to be found in the sheet ‘Figure5A_ttest’ in the file S4_Data.xlsx. The False Discovery Rate corrected p-values for these tests are to be found in the sheet ‘Figure5A_pvalues’. The individual functional connectivity matrices for which these t-tests were performed are found in the uploaded file subcortcal_connectivity.mat.

**Figure 5B** – The data for these cortical surface figures are average (mean) of the t-test values for the thalamus_lh and thalamus_rh, which are to be found in the sheets ‘Figure5A_ttest’ and ‘Figure5A_pvalues’ in the file S4_Data.xlsx. Please note however, that the order of the parcels has been rearranged for plotting on the cortical surface.

**Figure 5C** – The correlations (r-coefficient and p-value) between the individual changes in total integration (17 network level) and the individual changes in thalamocortical connectivity for each of the 400 cortical parcels is to be found in the sheet ‘Figure5C_correlations’ in the file S4_Data.xlsx.

**Figure 5D** – The individual values for the average (mean) connectivity between the thalamus and all 400 cortical parcels is to be found in the sheet ‘Figure5D_raw’ in the file S4_Data.xlsx.
