# Supplementary material for: An altered balance of integrated and segregated brain activity is a marker of cognitive deficits following sleep deprivation
Source: PLoS Biol. 2021 Nov 4;19(11):e3001232. doi: 10.1371/journal.pbio.3001232 (PMC8568176; doi:10.1371/journal.pbio.3001232)
Supplement: Attachment — Submitted filename: Response to Reviewers.docx [file pbio.3001232.s006.docx]

Reviewer #1: The authors examined the impact of sleep and sleep deprivation on whole brain functional integration. Sleep deprivation increased neural integration within networks, a trend that continued into NREM sleep, where neural integration was further accompanied by a decrease in between-network integration. Moreover, changes in task performance were strongly associated with altered neural integration following sleep loss as well as their reset following a nap. The study therefore suggests that changes in the dynamics of functional integration in the brain reflect an underlying neural correlate of cognitive impairment triggered by sleep loss.

The study is well designed. The authors carefully and comprehensively examined various measures of neural integration across a spectrum of arousal states, highlighting the shifts in intrinsic functional connectivity induced by sleep and sleep loss. I only have a few minor comments that can strengthen the findings and help clarify the results:

We would like to thank the reviewer for their positive feedback and comments on our study.

1)      In the abstract the authors mention that FCR serves as a "better marker of cognitive impairment than conventional indicators of homeostatic sleep pressure, as well as the pronounced thalamo-cortical connectivity changes that occurs towards falling asleep". However, there is no mention of an analysis examining the association between behavioral performance and conventional indicators of homeostatic sleep pressure. Can the authors please add?

We thank the reviewer for pointing out this omission! We have added this to the main manuscript (Page 7):

“*Furthermore, none of the aforementioned measures of homeostatic sleep pressure were related to the change in accuracy or reaction time from RW to SD (SI Results)*.”

and to the supplementary results sections (Page 10):

“*Relationships Between Performance and Homeostatic Markers of Sleep Pressure*

*There were no significant associations between the change in general performance on cognitive tasks with any marker of sleep homeostatic pressure. Sleep latency was not associated with change in accuracy score from RW to SD (r = 0.20, p = 0.417) or change in reaction time (r = -0.29, p = 0.224). Latency to NREM3 was not associated with change in accuracy score from RW to SD (r = 0.25, p = 0.346) or change in reaction time (r = -0.09, p = 0.718). Additionally, percentage of NREM Stage 3 during the nap was not associated with change in accuracy score from RW to SD (r = -0.24, p = 0.322) or change in reaction time (r = 0.24, p = 0.327). Finally, the change from RW to SD in the power of EEG frequencies 1-10Hz were not related to the change in accuracy from RW to SD (r values = -0.12-0.13, all p>0.613) or change in reaction time (r values = -0.21-0.13, all p>0.424)*.”

2)      Supplementary analyses of community structures and task evoked data (Tables S7 & S6) are mentioned in the main text but more details are missing. For instance- how do these findings support the main analyses in the paper? In what ways do they differ?

These additional analyses are primarily meant to serve as a control for the choices made in our analyses (e.g. regressing out task evoked responses, choice of parcellation template), and we wanted to show that the results are not dependent on these choices. We have added to the results section describing how these compare to the main findings (Page 5):

“*These analyses support the main findings. However, more cortical networks demonstrated significant increases in integration induced by SD when the task evoked activity were not regressed out (7 of the Yeo7 networks, and 15 of the Yeo17 networks), suggesting that task activations may interact with sleep deprivation to further drive an increase in cortical network integration*.”

It is difficult to compare the results obtained using the Yeo7 network parcellation template and the data-driven community structure, as the lower levels of the hierarchical model contain different regions. However, the fact that integration (total and within-network) increases from RW to the SD condition using both templates demonstrates that this observation is robust to the choice of parcellation used.

3)      In Figure 4D I would suggest renaming the Y axis to "whole brain thalamocortical connectivity" and adding indications for any arousal states that are significantly different from zero.

Figure 4D has now been updated with these suggested changes.

4)      Supplementary tables are missing a legend to explain the significance columns (p>0.95)- please clarify which tests were conducted in each table.

This has been updated for each table, with a brief explanation of how significance was determined: “^†^*Approximated posterior probability p(A|y) of the assertion that one state is greater than the other (e.g. FCR_SD_ > FCR_RW_). Calculated by as the frequency of the increase observed in the Bayesian sampling scheme (see Statistical Analysis).”*

5)      The analysis paragraph in the second page of the supplementary methods contains a spelling mistake "an global" should be " a global"

This has now been fixed. Thank you for the correction.

Reviewer #2: This is an interesting study showing that sleep deprivation increases the similarity to the sleeping brain, and that the similarity to wakefulness is restored after a nap. The authors used whole brain modularity of functional networks as a way to estimate these similarities. Interestingly, the changes in this measure also correlated with behavior and reflected fluctuations in global activity levels.

Here are some suggestions to improve the manuscript:

We would like to thank the reviewer for their time taken to review our article and their suggestions and comments.

1. The authors cite:

Boly, M., Perlbarg, V., Marrelec, G., Schabus, M., Laureys, S., Doyon, J., ... & Benali, H. (2012). Hierarchical clustering of brain activity during human nonrapid eye movement sleep. Proceedings of the National Academy of Sciences, 109(15), 5856-5861.

as an inspiration for some the analyses, but this paper contains analysis conducted during rest. I understand that the authors demonstrated sleep contamination during rest in this study, but wouldn't it be possible to salvage some epochs to replicate the findings in Boly et al., 2012, and include that in the main manuscript text?

We acknowledge that the functional analyses in the current paper are performed on data obtained during the performance of tasks, and not rest. While this task data is not the same as data obtained during the resting-state, we have focused the manuscript around the functional connectivity and integration changes during tasks as they relate to the simultaneous performance on these same tasks.

Unfortunately, given the nature of these analyses, it would be difficult to perform the analyses as the reviewer suggests using the same approach in calculating integration. Specifically, the covariance matrix becomes less reliable for shorter timeseries – especially if there is a large spatial dimension (i.e. 400 parcels). This would in turn make it difficult to obtain reliable measures of integration during such very short ‘rest’ epochs (ANT = 1-6 TRs; N-back = 4 TRs; PVT = 6 TRs, for comparison the resting state session is 120 TRs).

Nevertheless, our results, even though they were observed during the performance of tasks and not rest, still follow the same patterns as Boly et al. (during rest). In our opinion, this not only demonstrates partial replication of the findings from Boly, but further shows that they generalise to task fMRI. This also reinforces the interpretation that the integration/segregation balance is a marker of the level of consciousness, regardless of whether an individual is in the resting-state or performing tasks.

In our discussion, we mention this in separate occasions:

(Page 7): “*These changes were observed during the performance of cognitive tasks, and importantly were strongly related to overall performance*.”

(Page 8): “*We detected the greatest FCR during the NREM recovery nap (Fig 2C), consistent with previous observations during NREM sleep (Boly et al. 2012, Del Felice et al. 2015, Tagliazucchi et al. 2013). Although previous comparisons of NREM sleep were with the wakeful resting state, the current findings demonstrate that these effects generalise to other states of wakefulness (i.e. task performance)*.”

2. In Fig. 1C the networks and sub-networks cannot be appreciated due to poor contrast

We have changed the contrast on this figure to increase visibility of results.

3. Can you check the correlations using Spearman's correlation? some scatterplots show that linear correlations could be driven by outliers

The linear rank (Spearman’s) correlations reveal that all the correlations remain significant:

Accuracy, RW -> SD: rho = -0.71, p <0.0001

Reaction Time, RW -> SD: rho = 0.52, p = 0.019

Accuracy, SD -> PRN: rho = 0.63, p = 0.004

Reaction Time, SD -> PRN: rho = -0.68, p = 0.001

4. Boly et al. is not the only manuscript to investigate modularity during sleep, you could also cite
Del Felice, A., Storti, S. F., & Manganotti, P. (2015). Sleep affects cortical source modularity in temporal lobe epilepsy: a high-density EEG study. Clinical Neurophysiology, 126(9), 1677-1683.
Tagliazucchi, E., von Wegner, F., Morzelewski, A., Brodbeck, V., Jahnke, K., & Laufs, H. (2013). Breakdown of long-range temporal dependence in default mode and attention networks during deep sleep. Proceedings of the National Academy of Sciences, 110(38), 15419-15424.

We referenced Boly et al as the main reference as we took the same analysis method to calculate the (information theoretic) integration metrics as used in that paper. However, we have now added these additional references to the manuscript.

5. Are there interesting changes in EEG power that could also indicate increases similarity to sleep (e.g. slowing down) that could be correlated with the modularity results?

We do find a slight increase in the power of slower EEG frequencies (1-4Hz) from the RW to SD conditions (t-statistics = 2.1-2.3, p<0.05 uncorrected), which is consistent with the prior sleep deprivation literature. However, these were not related to any of the observed changes in either total integration or FCR.

This is now reported in both the main text (Page 7):

“*Additionally, despite a slight slowing of EEG frequencies from RW to SD (1-4Hz, SI Results), these changes were not related to the change in FCR or (I_Tot)*.”

And in the supplementary results (Page 10):

“*Secondly, the change from RW to SD in the power of EEG frequencies 1-10Hz were not related to the change in total integration (r values = 0.10-0.39, all p>0.129) or FCR (r values = -0.01-0.21, all p>0.413)*.”

Reviewer #3:

Summary:

The manuscript of Cross et al utilizes a simultaneous EEG-fMRI data to address the very interesting topic on the balance of brain activity integration/segregation following sleep deprivation and its effect in cognitive decline. They used methodologies that were previously introduced in the field to define measures of integration and segregation. They found that brain dynamics during sleep deprivation is characterized by an increased in global functional integration as driven by the rise of within cortical network integration. These metrics are then used to investigate the relationship to task performance following either the relaxed wakefulness, sleep deprived state, and post-recovery nap.

Overall, the manuscript is very interesting and provides strong evidence regarding the effective use of altered balance between integrated and segregated brain activity as a biomarker for cognitive performance following sleep deprivation. The paper also addressed many different issues that evaluates the robustness of the outcomes, making their conclusions sounder and more intact. The manuscript is written in a straightforward manner. However, it suffers from major setbacks in terms of the clarity of information given, especially in the methodological details. There are several points that the authors need to address, in particular, with regards to the strong assumptions made in the model as well as the consistency of the terminologies used in the manuscript.

We would like to thank the reviewer for their review of our article, and their constructive feedback. We have done our best to address all their comments and incorporate them into the manuscript.

Major Comments

1.      It's not clear how you define integration and total integration. It is sometimes confusing to which level in the hierarchy the authors refer to when they mention the word integration or total integration in the text. I suggest that they put these terms into specific variables or notations so it is much clearer to understand. These notations can be found in the supplementary, and only becomes clearer if you specifically look for it. Consider moving those to the manuscript itself.

We understand how this may have been unclear and confusing, and we appreciate the reviewer’s feedback in highlighting this in our description of the methodology. We have moved a substantial proportion of our methodology from the supplementary materials to the main manuscript (Methods) and hope this is easier to access for readers’ understanding.

Furthermore, we explicitly define total integration as the integration (within + between) concerning the entire cortex, and denote it as ($I_{Tot}$). For all other integration metrics (ie. total integration within specific networks), we just use the term integration.

2.      The computation of the integration assumes that the fMRI BOLD data associated with the regions selected are temporally independent and identically distributed (i.i.d). However, there is an inherent sample dependence in the spatial or temporal dimension of the fMRI data, which therefore violates the iid assumption. How valid is this assumption? What specific measures were taken to mitigate the effects of such a strong assumption? Also, smoothing of the fMRI data minimize the impact of high frequency noise in fMRI and also reduces the amount of spatial variability across subjects. How does the smoothing of the data (in this case 6mm) affect the strong iid assumption on the nature of the time-series you are considering? How does this affect the results?

We appreciate this very insightful comment from the reviewer.

Indeed, the measurement of integration is based on three important assumptions:

1. The distribution of fMRI data is Gaussian. This hypothesis has already been discussed in the literature, in particular on the use of the GLM model, and seems valid in the case where the data are smoothed with a Gaussian spatial filter (Worsley & Friston, 1995). A 6mm filter is used in our study.

2. The fMRI data are spatially independent. Clearly this hypothesis is not valid and several models have been proposed to take into account the effect of spatial correlation in the fMRI results. However, it has been shown that the effect of the spatial correlation is important at the small spatial scale (i.e. few voxels) and is less important at the regional level (Bellec et al. 2006, Kiebel et al. 2007). In Bellec et al. (page 1236), the (spatial) distance where the spatial correlation effect is important is about 30mm at most. For region sizes smaller than 30mm, the independence assumption in the integration calculation (ie. Independence model) is probably not valid.

We do not use raw voxels in 3D fMRI volume space. Instead, we have sampled the BOLD signal along the cortical mantle. Then, we use a standardised parcellation template that has been developed using a gradient-weighted Markov Random Field (gwMRF) model integrating local gradient and global similarity approaches (Schaeffer et al. 2018), which further reduces the spatial dependencies in the data. Finally, with regards to the spatial smoothing of 6mm, the parcels from this template spans multiple 2x2x2mm voxels (in the volume space), all of which are > 6mm:

*(Taken from Schaefer et al, 2018)*

The size of the regions we have considered in our study is greater than 30mm. Therefore, the effect of spatial correlation would be negligible.

3. The fMRI data are temporally independent. fMRI data exhibit short-range temporal correlations. By this we mean that the error at a given scan is correlated with its temporal neighbours. This has to be modelled, because ignoring correlations leads to an inappropriate estimate of the degrees of freedom and statistics (Kiebel et al. 2007). With serial-temporal correlations the effective degrees of freedom are lower than in the case of independence. So, we will therefore bias the calculation of the temporal covariates and consequently the estimation of the statistical thresholds of the integration measures. We would like to emphasise that integration is the quotient of the determinants of two covariance matrices. This bias is as small as the number of time points in the calculation of the integration is high (i.e. asymptotic assumption). In our sleep study the time series had 624 time points. However, it would be relevant to study the robustness (as a function of the size of the ROIs and the number of time points) of the sampling procedure we use to determine the posterior distribution of the integration. This study is well beyond our practical considerations in this article.

Finally and importantly, any inherent dependencies in the timeseries should be comparable within subjects across conditions. Thus, even if integration were slightly overestimated, due to our repeated measures design of measuring the same individuals multiple times, we would expect either of two scenarios: a) uniformly overestimated integration in each condition, or b) the greater integration that we observe in the SD condition would be reflected as greater sample (temporal) dependencies in the BOLD signal. This latter point should be investigated in a more detailed analysis of functional dynamics (e.g. dynamic FC, fluctuations in statistical dependencies of regions over time) in the SD state.

We do not believe this violates our main findings that *changes in integration* from the RW to the SD are related to changes in cognitive function. Rather, this changes the interpretation of potential underlying causes of a change in integration in the SD state. We have incorporated this in the discussion of our manuscript (Page 9):

“*Finally, given the assumptions of our model of integration, in particular that the BOLD data at each time point are temporally independent and identically distributed (i.i.d) realizations of a 400-dimensional random variable y (Marrelec et al, 2008), it is possible that potential sample dependencies across time could contribute to an overestimation of integration measures within the data. This could mean that the elevated integration we observed in the SD condition are due to an increase in temporal dependencies in the data. This should be investigated in future studies investigating the dynamics of brain activity in the SD state. The effect of spatial dependence in the BOLD timeseries would have had a negligible impact on these findings, as it has been shown that this effect is important at the small spatial scale (i.e. few voxels) and is less important at the regional level (ie. >30mm), the latter of which was implemented in this study (Bellec et al., 2006).*”

3.      The manuscript applied different methods in a successive manner, which can be very confusing at first reading. Consider providing a figure that clearly illustrate step by step methods applied (methodological paradigm).

We have combined supplementary Figures 1 and 2 into a new Figure to be included in the main text (now Figure 1), which we hope will help in clarifying our methodological steps.

We have also greatly expanded the methods section in the main text to aid with this understanding as well.

4.      On the PCA analysis of accuracy and speed, explain clearly what is included in the data matrix (e.g., #subjects x # task metrics). Why is there a need to perform PCA analysis to obtain a global measure of performance? What are the outcomes if you used raw values of accuracy and speed in assessing the relationship of task performance and brain segregation/integration measures?

In the expanded methods section, we describe the PCA analyses in more detail (Page 10):

“*The change in performance outcomes on each of the 3 separate cognitive tasks were grouped into either accuracy (% of correct responses) or speed (reaction time, in ms). This provided 2 data matrices of size #subjects x #tasks; one matrix containing accuracy scores and one containing reaction times. The 3 change scores for accuracy or speed were entered into separate Principal Component Analyses (PCA), in order to provide a global estimate of performance. The first principal component from each PCA was extracted and used as a single measure for Accuracy or Speed across the 3 tasks.*”

The rationale for using PCA here is twofold:

1) it provides a more general measure of cognitive impairment induced by sleep deprivation, not specific to performance on an individual task or in a particular domain;

2) it reduces the dimensions (ie. number of tests) for probing associations between integration and performance.

Nevertheless, we still report the associations between raw scores on each task and integration measures in the main text (Page 6):

“*We also assessed the change in performance on each task separately in comparison to the change in integration of cortical BOLD activity during that task only. A greater increase in integration was significantly related to worse accuracy and speed in the ANT and N-back tasks, but not the MCT (Fig. S7).*”

5.      How many subjects reached N3? Was it difficult or easy for subjects to fall asleep and reach deep levels of sleep? You provided the amount of recorded sleep in each stage in terms of percentage - but how many participants actually reached N2 and N3?

It was reasonably easy for subjects to fall asleep, with an average sleep latency of 5 minutes. During the nap, 16/19 (84%) of subjects achieved NREM3 sleep. However, only 11/19 (58%) of subjects were able to maintain stable periods of NREM3 sleep (>5mins). This demonstrates that it was difficult to maintain deep sleep in the MRI environment, even though the sleep pressure was high. This is not surprising however, as the MRI environment in combination with a high-density EEG cap are disruptive to stable and deep sleep. All 19 subjects who managed to sleep reached stable N2 sleep (>5min). We have added these numbers to Supplementary Table S8.

6.      In the analysis of post-recovery nap, why did you consider only N2 and N3, and not N1? Did you also look at the metrics during wakefulness (before the participants fell asleep in the scanner)?

We only considered N2 and N3 in our analysis of the recovery nap for multiple reasons:

1) these are deeper sleep and have been shown to be more important sleep stages than N1 (N1 being much reduced in recovery sleep) for the recovery of homeostatic sleep pressure and the cognitive deficits of sleep deprivation (Jung et a, 2011; Ong et al, 2016);

2) these N2 and N3 sleep stages are more stable than N1, and we were able to extract continuous segments of N2 & N3 with only 1 stage shift;

3) the scoring of stages N2 and N3 sleep is much more reliable and discernible from wakefulness than N1 on EEG-fMRI due to the noisy environment, impacting the quality of more subtle EEG differences.

The average sleep latency after the start of the scanning during the nap sequence was 5 min (with 13/19 subjects sleep latency < 1 min; Table S8), which was expected due to the high sleep pressure following total sleep deprivation. Therefore, it was not possible to extract any reliable measures of FC or integration during this short time period of wakefulness across subjects. However, given the study design, the tasks during the SD condition were performed immediately prior to the nap, so they would capture very similar BOLD activity as would the few minutes before subjects fell asleep.

7.      Would it be interesting to look at the changes in brain signal integration/segregation across NREM sleep stages? How is it different for sleep deprived and well-rested individuals? Do you think there will be some differences?

We appreciate the reviewer’s interest in this area. This was not the focus of our paper to investigate differences in integration within the sleep period. However we are currently investigating this in another study, using fewer parcellations to compare resting state (well-rested) and sleep data. In our discussion we have speculated upon this matter based on prior findings from the literature using similar metrics of mutual information between BOLD timeseries (Page 8):

“*We were not able to robustly measure the differences between NREM2 and NREM3 in this study with the same amount of consecutive data points as in the wake conditions, however other studies suggest that brain networks are even more segregated during NREM3 compared to NREM2 sleep (Tarun et al. 2021), and that total integration decreases in proportion to SWA (Boly et al. 2012)*.”

Finally, it would be an interesting question to compare regular sleep compared with recovery sleep following SD and assess whether there is increased functional clustering in recovery sleep. This was not possible given the design of the study (no nap was attempted in the WR condition due to low sleep pressure and thus likelihood of sleep). Recovery sleep usually contains more slow wave sleep with greater slow wave activity. Recent studies (e.g. Ong et al. 2019) suggest that regular sleep and recovery sleep following SD share very stable, trait-like spectral properties in EEG, with the exception of lower frequencies. Given these facts, and the fact that the FCR increases in NREM3 (SWS), we would hypothesise that the FCR would be slightly greater in recovery sleep compared to sleep when it is not followed by SD. However further studies would need to be designed specifically to answer this question.

8.      In computing functional connectivity, why did you z-score the correlation matrices? Would it make more sense to z-score the activity time-courses directly?

This is a relatively routine step in functional connectivity analyses across the literature (e.g. Yeo et al 2015, Cohen & D’Esposito 2016, Finn et al 2015, amongst others). As per the nature of correlations, the series of correlation coefficients generated in a functionally connectivity matrix usually exhibit a skewed distribution across all edges. In order for use in parametric testing (e.g. t-tests, anovas), z-scoring the correlation coefficients renders a normal distribution of correlation coefficients. Z-scoring the timeseries would have no effect on the Pearson correlation between parcels themselves, unless the timeseries from all subjects and sessions were first concatenated and then z-scored, which would be quite computationally intensive. Z-scoring the connectivity matrices themselves ensures normality of correlation values without having to go to this extreme.

With regards to the main analyses (integration), the correlation matrices were not used, instead these measures were derived from the covariance matrices.

9.      The authors evaluated the differences in the amplitude of global signal fluctuations in each condition, and have shown that regressing out the motion does not change the results. How did you compute this? When you mean fluctuation, did you mean that you assessed the variance of the BOLD amplitude within each condition?

We apologise for any confusion. We did not regress out motion in our comparison of global signal fluctuation between states (although motion was regressed out of the BOLD signal in the preprocessing). Rather, we demonstrated that the relationship between total integration ($I_{Tot}$) and the global signal fluctuation remained significant when motion was included as a covariate in the regression model per state. This was performed with a standard GLM.

However, in order to address the reviewer’s comment, we reran the comparison analyses of global signal fluctuation between conditions, after frame displacement (FD) was regressed out from the timeseries, in the same manner as we did for regressing out the task-blocks (ie. taking the residuals from the regression model). The increase in global signal fluctuation remained significant (t = 3.75, p<0.01).

Finally, we define this fluctuation in the results section (Page 7):

“… *the amplitude of the global signal fluctuation (standard deviation of the global signal time series)*”

10.     According to the differences in task performance following different conditions (WR, SD, and PRN), there are no differences in the performance of individuals following WR or PRN (Fig S1 C), even though there is a huge discrepancy of the length of time that these individuals rested and slept in each condition. How do you think the results in task performance would play out as we increase the waiting time between PRN and the actual task activity?

This is an interesting question. Indeed, it is a curious observation that performance returns to levels that are not different from baseline after just 60 minutes (approx.) of sleep. Particularly given that integration levels do not return to normal (on average) after the nap. However, we do observe that those whose functional clustering ratio decreases the most following the nap have the greatest recovery of performance. This suggests that the restoration of functional integration/segregation may be more important than total sleep time. Additionally, while there were differences in the total sleep time across subjects, we do not think that there is a huge discrepancy (50 ± 12 minutes).

If the time between the end of the nap and the PRN session (task performance) was increased, there may indeed be a build-up of sleep pressure again, which could impact task performance. However, subjects may also experience sleep inertia, which would also dissipate the longer the waiting time after the nap. Sleep inertia may also negatively impact performance, so we expect there is probably a trade-off between these factors in their influence on PRN task performance.

11.     In computing for integration in subsystems, how do you account for the differences in the number of subsystems that are present in each network?

Unfortunately, we cannot account for the number of subsystems in each network in our Bayesian inference like we could in a GLM. However, to address the reviewer’s comment, we have explored this further.

Firstly, we correlated the total integration within each of the Yeo17 networks with the number of assemblies in that network. We found no relationship between the total integration and the number of assemblies, within any of the conditions RW (r=-0.18,p=0.47), SD (r=-0.20, p=0.43) or PRN (r=-0.12, p=0.63). Furthermore, the absolute change in integration from RW to SD was not related to the number of assemblies within each of the Yeo17 networks (r=-0.22, p=0.38).

Minor Comments

1.      There is an inconsistent (or confusing) use of relaxed wakefulness (RW) vs well-rested (WR) acronyms. Some parts of the manuscript use RW and on others, it's WR. Please ensure consistency of word usage in the whole manuscript. Are they pertaining to the same thing?

We have reviewed all cases of these acronyms in the manuscript and have ensured all acronyms pertaining to the Rested Wakefulness session are ‘RW’.

2.      The figures are too small and are squeezed together. Please increase the size of the figure and the corresponding font of the texts.

We have updated the figures, particularly Figure 3 (now Figure 4), to increase the size and make the results clearer. We have increased the font as much as possible.

3.      Some parts of the text point to different figures in the supplementary --  [e.g., "Following the SD condition, 19 of the 20 participants managed to successfully sleep inside the scanner, for an average of 50 ± 12 minutes (a sleep summary can be found in Table S7)"  -- Table S8 rather].

We have reviewed all references to figures and tables in the manuscript and ensured these are now correct.

References:

1. Ong JL, Lo JC, Gooley JJ, Chee MW. EEG Changes across Multiple Nights of Sleep Restriction and Recovery in Adolescents: The Need for Sleep Study. Sleep. 2016 Jun 1;39(6):1233-40. doi: 10.5665/sleep.5840. PMID: 27091536; PMCID: PMC4863211.
2. Ong JL, Lo JC, Patanaik A, Chee MWL. Trait-like characteristics of sleep EEG power spectra in adolescents across sleep opportunity manipulations. J Sleep Res. 2019 Oct;28(5):e12824. doi: 10.1111/jsr.12824. Epub 2019 Feb 6. PMID: 30724415; PMCID: PMC6899593.
3. Marrelec G, Bellec P, Krainik A, Duffau H, Pélégrini-Issac M, Lehéricy S, Benali H, Doyon J. Regions, systems, and the brain: hierarchical measures of functional integration in fMRI. Med Image Anal. 2008 Aug;12(4):484-96. doi: 10.1016/j.media.2008.02.002. Epub 2008 Feb 15. PMID: 18396441.
4. Jung, C.M., Melanson, E.L., Frydendall, E.J., Perreault, L., Eckel, R.H. and Wright, K.P. (2011), Energy expenditure during sleep, sleep deprivation and sleep following sleep deprivation in adult humans. The Journal of Physiology, 589: 235-244. <https://doi.org/10.1113/jphysiol.2010.197517>
5. P Bellec, V Perlbarg, S Jbabdi, M Pelegrini-Issac, J.L. Anton, J. Doyon, and H. Benali. Identification of large-scale networks in the brain using fMRI. NeuroImage 29 (2006) 1231 – 1243.
6. F. Kruggel, M. Pelegrini-Issac, M., H. Benali. Estimating the effective degrees of freedom in univariate multiple regression analysis. Med. Image Anal. 2002, 6, 63– 75.
7. Worsley, K.J., Friston, K.J., 1995. Analysis of fMRI time-series revisitedagain, NeuroImage 2, 173– 181.
8. S.J. Kiebel, A.P. Kherif and C. Holmes. The general linear model. In Statistical Parametric Mapping, by Karl Friston et al. ISBN–13: 978-0-12-372560-8 ISBN–10: 0-12-372560-7, 2007, Pages 101-125
