# Supplementary material for: An altered balance of integrated and segregated brain activity is a marker of cognitive deficits following sleep deprivation
Source: PLoS Biol. 2021 Nov 4;19(11):e3001232. doi: 10.1371/journal.pbio.3001232 (PMC8568176; doi:10.1371/journal.pbio.3001232)
Supplement: Peer review history

# Editor report: Decision Letter 0

**DOI:** 10.1371/journal.pbio.3001232.r001

**Authors:** Gabriel Gasque

20 Apr 2021

Dear Dr Cross, 

Thank you for submitting your manuscript entitled "An altered balance of integrated and segregated brain activity is a marker of cognitive deficits following sleep deprivation" for consideration as a Research Article by PLOS Biology.

Your manuscript has now been evaluated by the PLOS Biology editorial staff, as well as by an academic editor with relevant expertise, and I am writing to let you know that we would like to send your submission out for external peer review.

However, before we can send your manuscript to reviewers, we need you to complete your submission by providing the metadata that is required for full assessment. To this end, please login to Editorial Manager where you will find the paper in the 'Submissions Needing Revisions' folder on your homepage. Please click 'Revise Submission' from the Action Links and complete all additional questions in the submission questionnaire.

Please re-submit your manuscript within two working days, i.e. by Apr 22 2021 11:59PM.

Login to Editorial Manager here: https://www.editorialmanager.com/pbiology

During resubmission, you will be invited to opt-in to posting your pre-review manuscript as a bioRxiv preprint. Visit http://journals.plos.org/plosbiology/s/preprints for full details. If you consent to posting your current manuscript as a preprint, please upload a single Preprint PDF when you re-submit. 

Once your full submission is complete, your paper will undergo a series of checks in preparation for peer review. Once your manuscript has passed all checks it will be sent out for review. 

Given the disruptions resulting from the ongoing COVID-19 pandemic, please expect delays in the editorial process. We apologise in advance for any inconvenience caused and will do our best to minimize impact as far as possible.

Feel free to email us at plosbiology@plos.org if you have any queries relating to your submission.

Kind regards,

Gabriel Gasque, Ph.D.,

Senior Editor

PLOS Biology

# Peer review: Decision Letter 1

**DOI:** 10.1371/journal.pbio.3001232.r002

**Authors:** Gabriel Gasque

2 Jun 2021

Dear Dr Cross,

Thank you very much for submitting your manuscript "An altered balance of integrated and segregated brain activity is a marker of cognitive deficits following sleep deprivation" for consideration as a Research Article at PLOS Biology. Your manuscript has been evaluated by the PLOS Biology editors, by an Academic Editor with relevant expertise, and by three independent reviewers. Please accept my apologies for the delay in sending the decision below to you.

In light of the reviews (below), we will not be able to accept the current version of the manuscript, but we would welcome re-submission of a much-revised version that takes into account the reviewers' comments. We cannot make any decision about publication until we have seen the revised manuscript and your response to the reviewers' comments. Your revised manuscript is also likely to be sent for further evaluation by the reviewers.

We expect to receive your revised manuscript within 3 months. 

Please email us (plosbiology@plos.org) if you have any questions or concerns, or would like to request an extension. At this stage, your manuscript remains formally under active consideration at our journal; please notify us by email if you do not intend to submit a revision so that we may end consideration of the manuscript at PLOS Biology.

**IMPORTANT - SUBMITTING YOUR REVISION**

Your revisions should address the specific points made by each reviewer. Having discussed these with the Academic Editor, we think you should pay special attention to reviewer 2's point 3 (i.e. correlations driven by outliers) and this reviewer's comments about missing references to similar previous studies. Please address thoroughly reviewer 3's methodological concerns. 

Please submit the following files along with your revised manuscript:

1. A 'Response to Reviewers' file - this should detail your responses to the editorial requests, present a point-by-point response to all of the reviewers' comments, and indicate the changes made to the manuscript. 

*NOTE: In your point by point response to the reviewers, please provide the full context of each review. Do not selectively quote paragraphs or sentences to reply to. The entire set of reviewer comments should be present in full and each specific point should be responded to individually, point by point.

You should also cite any additional relevant literature that has been published since the original submission and mention any additional citations in your response. 

2. In addition to a clean copy of the manuscript, please also upload a 'track-changes' version of your manuscript that specifies the edits made. This should be uploaded as a "Related" file type. 

*Re-submission Checklist*

When you are ready to resubmit your revised manuscript, please refer to this re-submission checklist: https://plos.io/Biology_Checklist

To submit a revised version of your manuscript, please go to https://www.editorialmanager.com/pbiology/ and log in as an Author. Click the link labelled 'Submissions Needing Revision' where you will find your submission record. 

Please make sure to read the following important policies and guidelines while preparing your revision:

*Published Peer Review*

Please note while forming your response, if your article is accepted, you may have the opportunity to make the peer review history publicly available. The record will include editor decision letters (with reviews) and your responses to reviewer comments. If eligible, we will contact you to opt in or out. Please see here for more details:

https://blogs.plos.org/plos/2019/05/plos-journals-now-open-for-published-peer-review/

*PLOS Data Policy*

Please note that as a condition of publication PLOS' data policy (http://journals.plos.org/plosbiology/s/data-availability) requires that you make available all data used to draw the conclusions arrived at in your manuscript. If you have not already done so, you must include any data used in your manuscript either in appropriate repositories, within the body of the manuscript, or as supporting information (N.B. this includes any numerical values that were used to generate graphs, histograms etc.). For an example see here: http://www.plosbiology.org/article/info%3Adoi%2F10.1371%2Fjournal.pbio.1001908#s5

*Blot and Gel Data Policy*

We require the original, uncropped and minimally adjusted images supporting all blot and gel results reported in an article's figures or Supporting Information files. We will require these files before a manuscript can be accepted so please prepare them now, if you have not already uploaded them. Please carefully read our guidelines for how to prepare and upload this data: https://journals.plos.org/plosbiology/s/figures#loc-blot-and-gel-reporting-requirements

*Protocols deposition*

To enhance the reproducibility of your results, we recommend that if applicable you deposit your laboratory protocols in protocols.io, where a protocol can be assigned its own identifier (DOI) such that it can be cited independently in the future. Additionally, PLOS ONE offers an option for publishing peer-reviewed Lab Protocol articles, which describe protocols hosted on protocols.io. Read more information on sharing protocols at https://plos.org/protocols?utm_medium=editorial-email&utm_source=authorletters&utm_campaign=protocols

Thank you again for your submission to our journal. We hope that our editorial process has been constructive thus far, and we welcome your feedback at any time. Please don't hesitate to contact us if you have any questions or comments.

Sincerely,

Gabriel Gasque

Senior Editor

PLOS Biology

ggasque@plos.org

*****************************************************

REVIEWS:

Reviewer #1: The authors examined the impact of sleep and sleep deprivation on whole brain functional integration. Sleep deprivation increased neural integration within networks, a trend that continued into NREM sleep, where neural integration was further accompanied by a decrease in between-network integration. Moreover, changes in task performance were strongly associated with altered neural integration following sleep loss as well as their reset following a nap. The study therefore suggests that changes in the dynamics of functional integration in the brain reflect an underlying neural correlate of cognitive impairment triggered by sleep loss. 

The study is well designed. The authors carefully and comprehensively examined various measures of neural integration across a spectrum of arousal states, highlighting the shifts in intrinsic functional connectivity induced by sleep and sleep loss. I only have a few minor comments that can strengthen the findings and help clarify the results: 

1) In the abstract the authors mention that FCR serves as a "better marker of cognitive impairment than conventional indicators of homeostatic sleep pressure, as well as the pronounced thalamo-cortical connectivity changes that occurs towards falling asleep". However, there is no mention of an analysis examining the association between behavioral performance and conventional indicators of homeostatic sleep pressure. Can the authors please add? 

2) Supplementary analyses of community structures and task evoked data (Tables S7 & S6) are mentioned in the main text but more details are missing. For instance- how do these findings support the main analyses in the paper? In what ways do they differ? 

3) In Figure 4D I would suggest renaming the Y axis to "whole brain thalamocortical connectivity" and adding indications for any arousal states that are significantly different from zero. 

4) Supplementary tables are missing a legend to explain the significance columns (p>0.95)- please clarify which tests were conducted in each table.

5) The analysis paragraph in the second page of the supplementary methods contains a spelling mistake "an global" should be " a global"

Reviewer #2: This is an interesting study showing that sleep deprivation increases the similarity to the sleeping brain, and that the similarity to wakefulness is restored after a nap. The authors used whole brain modularity of functional networks as a way to estimate these similarities. Interestingly, the changes in this measure also correlated with behavior and reflected fluctuations in global activity levels. 

Here are some suggestions to improve the manuscript:

1. The authors cite:

Boly, M., Perlbarg, V., Marrelec, G., Schabus, M., Laureys, S., Doyon, J., ... & Benali, H. (2012). Hierarchical clustering of brain activity during human nonrapid eye movement sleep. Proceedings of the National Academy of Sciences, 109(15), 5856-5861.

as an inspiration for some the analyses, but this paper contains analysis conducted during rest. I understand that the authors demonstrated sleep contamination during rest in this study, but wouldn't it be possible to salvage some epochs to replicate the findings in Boly et al., 2012, and include that in the main manuscript text?

2. In Fig. 1C the networks and sub-networks cannot be appreciated due to poor contrast

3. Can you check the correlations using Spearman's correlation? some scatterplots show that linear correlations could be driven by outliers

4. Boly et al. is not the only manuscript to investigate modularity during sleep, you could also cite

Del Felice, A., Storti, S. F., & Manganotti, P. (2015). Sleep affects cortical source modularity in temporal lobe epilepsy: a high-density EEG study. Clinical Neurophysiology, 126(9), 1677-1683.

Tagliazucchi, E., von Wegner, F., Morzelewski, A., Brodbeck, V., Jahnke, K., & Laufs, H. (2013). Breakdown of long-range temporal dependence in default mode and attention networks during deep sleep. Proceedings of the National Academy of Sciences, 110(38), 15419-15424.

5. Are there interesting changes in EEG power that could also indicate increases similarity to sleep (e.g. slowing down) that could be correlated with the modularity results?

Reviewer #3: 

Summary: 

The manuscript of Cross et al utilizes a simultaneous EEG-fMRI data to address the very interesting topic on the balance of brain activity integration/segregation following sleep deprivation and its effect in cognitive decline. They used methodologies that were previously introduced in the field to define measures of integration and segregation. They found that brain dynamics during sleep deprivation is characterized by an increased in global functional integration as driven by the rise of within cortical network integration. These metrics are then used to investigate the relationship to task performance following either the relaxed wakefulness, sleep deprived state, and post-recovery nap.

Overall, the manuscript is very interesting and provides strong evidence regarding the effective use of altered balance between integrated and segregated brain activity as a biomarker for cognitive performance following sleep deprivation. The paper also addressed many different issues that evaluates the robustness of the outcomes, making their conclusions sounder and more intact. The manuscript is written in a straightforward manner. However, it suffers from major setbacks in terms of the clarity of information given, especially in the methodological details. There are several points that the authors need to address, in particular, with regards to the strong assumptions made in the model as well as the consistency of the terminologies used in the manuscript.

Major Comments

1. It's not clear how you define integration and total integration. It is sometimes confusing to which level in the hierarchy the authors refer to when they mention the word integration or total integration in the text. I suggest that they put these terms into specific variables or notations so it is much clearer to understand. These notations can be found in the supplementary, and only becomes clearer if you specifically look for it. Consider moving those to the manuscript itself.

2. The computation of the integration assumes that the fMRI BOLD data associated with the regions selected are temporally independent and identically distributed (i.i.d). However, there is an inherent sample dependence in the spatial or temporal dimension of the fMRI data, which therefore violates the iid assumption. How valid is this assumption? What specific measures were taken to mitigate the effects of such a strong assumption? Also, smoothing of the fMRI data minimize the impact of high frequency noise in fMRI and also reduces the amount of spatial variability across subjects. How does the smoothing of the data (in this case 6mm) affect the strong iid assumption on the nature of the time-series you are considering? How does this affect the results?

3. The manuscript applied different methods in a successive manner, which can be very confusing at first reading. Consider providing a figure that clearly illustrate step by step methods applied (methodological paradigm).

4. On the PCA analysis of accuracy and speed, explain clearly what is included in the data matrix (e.g., #subjects x # task metrics). Why is there a need to perform PCA analysis to obtain a global measure of performance? What are the outcomes if you used raw values of accuracy and speed in assessing the relationship of task performance and brain segregation/integration measures?

5. How many subjects reached N3? Was it difficult or easy for subjects to fall asleep and reach deep levels of sleep? You provided the amount of recorded sleep in each stage in terms of percentage - but how many participants actually reached N2 and N3?

6. In the analysis of post-recovery nap, why did you consider only N2 and N3, and not N1? Did you also look at the metrics during wakefulness (before the participants fell asleep in the scanner)?

7. Would it be interesting to look at the changes in brain signal integration/segregation across NREM sleep stages? How is it different for sleep deprived and well-rested individuals? Do you think there will be some differences?

8. In computing functional connectivity, why did you z-score the correlation matrices? Would it make more sense to z-score the activity time-courses directly? 

9. The authors evaluated the differences in the amplitude of global signal fluctuations in each condition, and have shown that regressing out the motion does not change the results. How did you compute this? When you mean fluctuation, did you mean that you assessed the variance of the BOLD amplitude within each condition?

10. According to the differences in task performance following different conditions (WR, SD, and PRN), there are no differences in the performance of individuals following WR or PRN (Fig S1 C), even though there is a huge discrepancy of the length of time that these individuals rested and slept in each condition. How do you think the results in task performance would play out as we increase the waiting time between PRN and the actual task activity?

11. In computing for integration in subsystems, how do you account for the differences in the number of subsystems that are present in each network? 

Minor Comments

1. There is an inconsistent (or confusing) use of relaxed wakefulness (RW) vs well-rested (WR) acronyms. Some parts of the manuscript use RW and on others, it's WR. Please ensure consistency of word usage in the whole manuscript. Are they pertaining to the same thing?

2. The figures are too small and are squeezed together. Please increase the size of the figure and the corresponding font of the texts.

3. Some parts of the text point to different figures in the supplementary -- [e.g., "Following the SD condition, 19 of the 20 participants managed to successfully sleep inside the scanner, for an average of 50 ± 12 minutes (a sleep summary can be found in Table S7)" -- Table S8 rather].

# Author comment: Author response to Decision Letter 1

**DOI:** 10.1371/journal.pbio.3001232.r003

30 Jul 2021

Attachment Submitted filename: Response to Reviewers.docx Click here for additional data file.

# Peer review: Decision Letter 2

**DOI:** 10.1371/journal.pbio.3001232.r004

**Authors:** Gabriel Gasque

26 Aug 2021

Dear Dr Cross,

Thank you for submitting your revised Research Article entitled "An altered balance of integrated and segregated brain activity is a marker of cognitive deficits following sleep deprivation" for publication in PLOS Biology. I have now obtained advice from the original reviewers and have discussed their comments with the Academic Editor. 

Based on the reviews, we will probably accept this manuscript for publication, provided you satisfactorily address the data and other policy-related requests listed below my signature.

As you address these items, please take this last chance to review your reference list to ensure that it is complete and correct. If you have cited papers that have been retracted, please include the rationale for doing so in the manuscript text, or remove these references and replace them with relevant current references. Any changes to the reference list should be mentioned in the cover letter that accompanies your revised manuscript.

We expect to receive your revised manuscript within two weeks. 

To submit your revision, please go to https://www.editorialmanager.com/pbiology/ and log in as an Author. Click the link labelled 'Submissions Needing Revision' to find your submission record. Your revised submission must include the following:

-  a cover letter that should detail your responses to any editorial requests, if applicable, and whether changes have been made to the reference list

-  a Response to Reviewers file that provides a detailed response to the reviewers' comments (if applicable)

-  a track-changes file indicating any changes that you have made to the manuscript. 

NOTE: If Supporting Information files are included with your article, note that these are not copyedited and will be published as they are submitted. Please ensure that these files are legible and of high quality (at least 300 dpi) in an easily accessible file format. For this reason, please be aware that any references listed in an SI file will not be indexed. For more information, see our Supporting Information guidelines:

https://journals.plos.org/plosbiology/s/supporting-information

*Published Peer Review History*

Please note that you may have the opportunity to make the peer review history publicly available. The record will include editor decision letters (with reviews) and your responses to reviewer comments. If eligible, we will contact you to opt in or out. Please see here for more details:

https://blogs.plos.org/plos/2019/05/plos-journals-now-open-for-published-peer-review/

*Early Version*

Please note that an uncorrected proof of your manuscript will be published online ahead of the final version, unless you opted out when submitting your manuscript. If, for any reason, you do not want an earlier version of your manuscript published online, uncheck the box. Should you, your institution's press office or the journal office choose to press release your paper, you will automatically be opted out of early publication. We ask that you notify us as soon as possible if you or your institution is planning to press release the article.

*Protocols deposition*

To enhance the reproducibility of your results, we recommend that if applicable you deposit your laboratory protocols in protocols.io, where a protocol can be assigned its own identifier (DOI) such that it can be cited independently in the future. Additionally, PLOS ONE offers an option for publishing peer-reviewed Lab Protocol articles, which describe protocols hosted on protocols.io. Read more information on sharing protocols at https://plos.org/protocols?utm_medium=editorial-email&utm_source=authorletters&utm_campaign=protocols

Please do not hesitate to contact me should you have any questions.

Sincerely,

Gabriel Gasque, Ph.D.,

Senior Editor,

ggasque@plos.org,

PLOS Biology

------------------------------------------------------------------------

BLURB: Please provide a blurb, which will be included in our weekly and monthly Electronic Table of Contents, sent out to readers of PLOS Biology, and may be used to promote your article in social media. The blurb should be about 30-40 words long and is subject to editorial changes. It should, without exaggeration, entice people to read your manuscript. It should not be redundant with the title and should not contain acronyms or abbreviations.

------------------------------------------------------------------------

FIGURE TITLES: Please include in each legend a title, which should convey the take-home message of the results shown in the figure. 

------------------------------------------------------------------------

ETHICS STATEMENT:

-- Please indicate within your manuscript if the written consent given by the participants was also informed.

-- Please indicate within your manuscript if your protocols approved by the Comité central d'éthique de la recherche (CCER) in Quebec adheres to the principles expressed in the Declaration of Helsinki or any other specific national or international ethical guidelines (which one?).

------------------------------------------------------------------------

DATA POLICY:

You may be aware of the PLOS Data Policy, which requires that all data be made available without restriction: http://journals.plos.org/plosbiology/s/data-availability. For more information, please also see this editorial: http://dx.doi.org/10.1371/journal.pbio.1001797

PLOS recognizes that, in some instances, authors may not be able to make their underlying data set publicly available for legal or ethical reasons. This data policy does not overrule local regulations, legislation or ethical frameworks. If there is an existing framework that prevents or limits release of your data, please make these limitations clear in the Data Availability Statement. For acceptable restrictions on public data sharing please read this link: https://journals.plos.org/plosbiology/s/data-availability#loc-acceptable-data-access-restrictions

However, please dote that we do not require all raw data. Rather, we ask for all individual quantitative observations that underlie the data summarized in the figures and results of your paper. For an example see here: http://www.plosbiology.org/article/info%3Adoi%2F10.1371%2Fjournal.pbio.1001908#s5

These data can be made available in one of the following forms:

1) Supplementary files (e.g., excel). Please ensure that all data files are uploaded as 'Supporting Information' and are invariably referred to (in the manuscript, figure legends, and the Description field when uploading your files) using the following format verbatim: S1 Data, S2 Data, etc. Multiple panels of a single or even several figures can be included as multiple sheets in one excel file that is saved using exactly the following convention: S1_Data.xlsx (using an underscore).

2) Deposition in a publicly available repository. Please also provide the accession code or a reviewer link so that we may view your data before publication.

Regardless of the method selected, please ensure that you provide the individual numerical values that underlie the summary data displayed in the following figure panels: Figures 1D, 2A-C, 3A-C, 4A-F, and 5A-D.

NOTE: the numerical data provided should include all replicates AND the way in which the plotted mean and errors were derived (it should not present only the mean/average values).

Please also ensure that each figure legend in your manuscript includes information on where the underlying data can be found and that your supplemental data file/s has/have a legend.

Please ensure that your Data Statement in the submission system accurately describes where your data can be found.

------------------------------------------------------------------------

Reviewer remarks:

Reviewer #1, Eti Ben Simon: The authors have addressed all my concerns. Congratulations on a wonderful study!

Reviewer #2: The authors have provided a satisfactory answer to all my comments and questions.

Reviewer #3, Anjali Tarun: The authors did a great job in revising their manuscript. They answered all my questions in detail.

# Editor report: Decision Letter 3

**DOI:** 10.1371/journal.pbio.3001232.r005

**Authors:** Gabriel Gasque

28 Sep 2021

Dear Nathan,

On behalf of my colleagues and the Academic Editor, Simon Hanslmayr, I am pleased to say that we can in principle offer to publish your Research Article "An altered balance of integrated and segregated brain activity is a marker of cognitive deficits following sleep deprivation" in PLOS Biology, provided you address any remaining formatting and reporting issues. These will be detailed in an email that will follow this letter and that you will usually receive within 2-3 business days, during which time no action is required from you. Please note that we will not be able to formally accept your manuscript and schedule it for publication until you have made the required changes.

As you address the formatting and reporting issues, please also:

(1) ensure that each figure legend in your manuscript includes information on where the underlying data can be found. For example, you can write: Data underlying this figure can be found in SX Data.

(2) ensure that your supplemental data folder has a legend. 

Please take a minute to log into Editorial Manager at http://www.editorialmanager.com/pbiology/, click the "Update My Information" link at the top of the page, and update your user information to ensure an efficient production process.

PRESS

We frequently collaborate with press offices. If your institution or institutions have a press office, please notify them about your upcoming paper at this point, to enable them to help maximise its impact. If the press office is planning to promote your findings, we would be grateful if they could coordinate with biologypress@plos.org. If you have not yet opted out of the early version process, we ask that you notify us immediately of any press plans so that we may do so on your behalf.

We also ask that you take this opportunity to read our Embargo Policy regarding the discussion, promotion and media coverage of work that is yet to be published by PLOS. As your manuscript is not yet published, it is bound by the conditions of our Embargo Policy. Please be aware that this policy is in place both to ensure that any press coverage of your article is fully substantiated and to provide a direct link between such coverage and the published work. For full details of our Embargo Policy, please visit http://www.plos.org/about/media-inquiries/embargo-policy/.

Thank you again for choosing PLOS Biology for publication and supporting Open Access publishing. We look forward to publishing your study. 

Sincerely, 

Gabriel Gasque, Ph.D. 

Senior Editor 

PLOS Biology

ggasque@plos.org
